# National Prescription Patterns of Antidepressants in the Treatment of Adults With Major Depression in the US Between 1996 and 2015: A Population Representative Survey Based Analysis

**DOI:** 10.3389/fpsyt.2020.00035

**Published:** 2020-02-14

**Authors:** Yan Luo, Yuki Kataoka, Edoardo G. Ostinelli, Andrea Cipriani, Toshi A. Furukawa

**Affiliations:** ^1^Department of Health Promotion and Human Behavior, School of Public Health in the Graduate School of Medicine, Kyoto University, Kyoto, Japan; ^2^Hospital Care Research Unit, Hyogo Prefectural Amagasaki General Medical Center, Hyogo, Japan; ^3^Department of Health Sciences, Università degli Studi di Milano, Milan, Italy; ^4^Department of Psychiatry, Warneford Hospital, University of Oxford, Oxford, United Kingdom

**Keywords:** major depressive disorder, antidepressant, prescription, trend, suboptimal dose

## Abstract

Few studies have delineated the real-world, long-term trends of prescription patterns of antidepressants for patients with major depressive disorder (MDD). This study aims to describe their vicissitudes in the nationally representative sample of the US from 1996 to 2015 and explore their characteristics. We used the Medical Expenditure Panel Survey, a nationally representative database of the US population, between 1996 and 2015. We estimated the prevalence of MDD among adults, calculated the proportions of those on antidepressant treatment as well as those on specific drugs through the two decades, and determined their dosages in 2015. We conducted multivariable regression to find possible factors related to their suboptimal prescriptions. The prevalence of adults diagnosed with MDD increased from 6.1% (95% CI, 5.7–6.6%) in 1996 to 10.4% (9.7–11.1%) in 2015. The proportion of patients without any antidepressant therapy decreased but still accounted for 30.6% (28.3–33.1%) in 2015. Sertraline and fluoxetine were among the most frequently prescribed antidepressants throughout the 20 years, while the trend for some new drugs changed dramatically. 16.1% (12.5–20.2%) of patients of MDD on antidepressant monotherapy were prescribed with suboptimal doses in 2015; the risk was lower for those who had higher Body Mass Index (OR 0.94 [0.90–0.99]), longer-term prescriptions (OR 0.92 [0.87–0.97]), and the risk was higher for those who were prescribed with tricyclic antidepressants (OR 11.21 [2.12–59.34], compared with serotonin reuptake inhibitors (SSRIs)), and antidepressants other than SSRIs and serotonin and norepinephrine reuptake inhibitors (OR 4.12 [1.95, 8.73], compared with SSRIs). This study confirmed the growing numbers of patients with MDD and the increase in the antidepressant prescriptions among them. However, the existence of patients without any antidepressant prescriptions or with suboptimal prescriptions and the variable prescription patterns through the decades might suggest some unresolved gaps between evidence and practice.

## Introduction

Depression is one of the most common mental disorders, with high prevalence in the population, resulting in impaired functions of affected individuals, then leading to great burden to the individuals and the society. Approximately 4.4% of the population (equivalent to more than 300 million people) in the world are estimated to suffer from depression in 2015, and the number is still increasing (1). In the United States, according to the National Survey on Drug Use and Health, about 7.1% of the adults had experienced at least one episode of major depressive disorder (MDD) in 2017, among which 63.8% had severe impairment (2, 3). In 2015, depressive disorders caused 7.5% of all Years Lived with Disability (YLD) globally, which ranked as the largest single contributor to non-fatal health loss world-wide (1). In the US, the incremental economic burden of individuals with MDD was $210.5 billion in 2010, which had increased by 21.5% since 2005 (4, 5).

Antidepressants play a key role in the treatment of MDD due to their demonstrated efficacy (6, 7) and wide availability. Monotherapy is recommended as the first-line initial treatment, while combination of antidepressants could also be considered if the initial monotherapy fails. In 1990s, as the effectiveness of different types of antidepressant appeared comparable, no specific recommendations were proposed by guidelines (8, 9). As many new generation antidepressants ushered into the market and as more and more evidence from randomized controlled trials (RCTs) have accumulated in the past three decades, practice guidelines in recent years started to give more specific recommendations regarding the classes or even within-class types of medications (10–12).

The details of actual prescriptions of antidepressants in the real world could then be very informative for the practitioners and the health policy makers in benchmarking their performances in depression treatment. Unfortunately, however, such details have not been well known, especially the population-based prescriptions of specific antidepressants targeting MDD and their changes over the time. Optimizing the doses of antidepressant should be equally crucial. A recent meta-analysis found a positive dose-response up to the lower end of licensed dose ranges of various antidepressants, beyond which there was no further increase in efficacy but only sharp increase in side effects (13). The average doses for particular antidepressants prescribed as monotherapy in treating MDD in the US and the potential factors related to their under-prescription remain unclear.

This study therefore aims to describe the national trends in the numbers of patients diagnosed with MDD, the characteristics of those who received antidepressant monotherapy, and the prescription patterns of individual antidepressants in treating MDD, through the past two decades between 1996 and 2015 based on a nationally representative survey database. We further estimated the daily average doses of frequently used antidepressants and explored the possible factors related to their suboptimal prescriptions.

## Materials and Methods

The protocol for this study has been published and is freely available (14). This study did not require institutional review board approval since only deidentified data were used. It was registered at UMIN Clinical Trials Registry (identifier: UMIN000031898).

### Sources of Data

We used the household components of the Medical Expenditure Panel Survey (MEPS) database (15). MEPS is a database sponsored by the Agency for Healthcare Research and Quality (AHRQ) and composed of yearly large-scale surveys of a representative sample of families and individuals and their medical providers, collecting data on the use of specific health services, the cost, and the health insurance in the United States since 1996. The participants were drawn from a subsample of households that participated in the prior year's National Health Interview Survey (NHIS). The sampling frame in MEPS gives a nationally representative sample of the non-institutionalized population in the US. Every year about 9,000 to 15,000 households, equivalent to 20,000 to 40,000 individuals are included. Data are collected using computer-assisted personal interview questionnaires, and every participant in one MEPS panel is interviewed by well-trained interviewers for five consecutive rounds within 2 years. Each participant is given a weight adjusting for nonresponse over time and some poststratification variables (region, race/ethnicity, sex, age, poverty status, etc.), in order to produce national estimates. (Further details of the MEPS surveys can be found in their webpage (15).

### Diagnosis of Depression

The MEPS collects information of diagnosis for each participant and codes them into 5-digit *International Classification of Diseases, Ninth Revision* (ICD-9) categories. The target population in this study were patients diagnosed with major depression, which had the corresponding ICD-9 code as 296.2 (major depressive disorder, single episode, 296.20–296.26), 296.3 (major depressive disorder, recurrent episode, 296.30–296.36), 311 (depressive disorder, not elsewhere classified). Patients with bipolar disorder were excluded. In order to use the detailed diagnostic information, this study has been approved by the AHRQ data center.

### Medications

In the MEPS database, each participant provided prescriptions of specific drugs, which were then confirmed by pharmacy providers when written permissions were provided. This study focused on the prescriptions of antidepressants, which have been approved for depression by the US Food and Drug Administration (FDA) and grouped them into 4 categories according to National Drug Code Directory (16): 1) Tricyclic Antidepressants (TCAs): amitriptyline, amoxapine, clomipramine, desipramine, doxepin, imipramine, nortriptyline, protriptyline, trimipramine; 2) Serotonin Reuptake Inhibitor (SSRIs): citalopram, escitalopram, fluoxetine, nefazodone, paroxetine, sertraline, trazodone; 3) Serotonin and Norepinephrine Reuptake Inhibitor (SNRIs): desvenlafaxine, duloxetine, venlafaxine, levomilnacipran; 4) No Pharm Class: bupropion, mirtazapine, vilazodone, vortioxetine. As described above, each participant received two or three rounds of interview within one year; in each interview the prescriptions only within that round were obtained. We defined patients on monotherapy as those who were prescribed with the same one antidepressant in all the rounds within that year, while those who were prescribed with different antidepressants within the same round or in different rounds within the same year were regarded as “patients receiving multiple antidepressants”. Dosages, including dose strength, quantity of prescribed medicine, and days of supplies in 2015 were also extracted, for the purpose of calculating the daily doses. A suboptimal prescription for each drug was defined as a dose lower than therapeutic range, which was according to the approved treatment doses for MDD by FDA (**Supplementary Table S1**).

Concomitant use of benzodiazepines, mood stabilizers and antipsychotics were extracted as well, for they were commonly used by major depressive patients. Based on FDA National Drug Code Directory, benzodiazepines included: alprazolam, chlordiazepoxide, clobazam, clonazepam, clorazepate, diazepam, estazolam, flurazepam, halazepam, lorazepam, midazolam, oxazepam, quazepam, temazepam, triazolam, zaleplon, and zolpidem; mood stabilizers included: carbamazepine, divalproex, lamotrigine, lithium, valproate and valproic acid. Antipsychotics included aripiprazole, asenapine, brexipiprazole, cariprazine, chlorpromazine, clozapine, fluphenazine, haloperidol, iloperidone, loxapine, lurasidone, molindone, olanzapine, paliperidone, perphenazine, pimavanserin, quetiapine, risperidone, thioridazine, thiothixene, and ziprasidone.

### Sociodemographic and Other Health-Related Characteristics

Sociodemographic information was collected for each participant, including age, sex, race/ethnicity, education level, marital status, family income level, health insurance. Body Mass Index (BMI) was also calculated in 2015. In this study, the target population was adults, aged 18 years or older.

Mental health status information was also available in the MEPS in 2015, as measured by Patient Health Questionnaire-2 (PHQ-2). Each participant was asked to complete the questionnaire during one interview in that year. The total score ranged from 0 to 6, and a cut-off of 3 was suggested by previous studies to be used as depression screening (17). The Kessler-6 Index (K6) was used to assess general psychological distress, with scores ranging between 0 and 24 and higher scores indicating higher level of distress in the past 30 days. Scores at 13 or more has been shown to indicate serious psychological stress (18, 19).

### Statistical Analyses

Data were extracted from the MEPS every five years from 1996 to 2015, i.e. in 1996, 2000, 2005, 2010 and 2015, since we considered that data at 5-year interval would be sufficiently fine-grained to show the trends in diagnoses and prescriptions. All the analyses were based on national estimates using sampling weights. The prevalence of major depressive disorder among adults was calculated for each year. The absolute numbers and percentages of depression patients who were receiving different kinds of treatment (no antidepressant treatment, antidepressant monotherapy or multiple antidepressants treatment) were presented for each year. For major depressive patients on antidepressant monotherapy, the trend of changes in sociodemographic characteristics, together with other health-related status, and the concurrent psychotropic treatments, were summarized over time. The prescription pattern of antidepressants as monotherapy was indicated by the number of patients being prescribed a specific drug and the proportion of patients on that drug among all the patients on monotherapy in each year. Since the survey methodology such as sampling and weighting and the measured items were being constantly improved over the years, directly comparing datasets from different times needs caution. Hence for this trend analysis, instead of employing statistical methods to give a *P* value, we opted rather to present the trends in a descriptive way.

We analyzed the doses of antidepressants prescribed as monotherapy for patients with MDD. The average daily doses for frequently prescribed antidepressants were estimated when the observed cases using a certain drug in the sample were more than 10. Crude odds ratios (ORs) with their 95% confidence intervals (CIs) were estimated for all the factors that may be associated with suboptimal use. We then used a model which adjusted age, sex and BMI for each variable to explore if the variable was potentially related to suboptimal prescriptions. Finally, we used a multivariable regression model to discover the factors that were strongly related to suboptimal prescriptions independently with each other based on available data.

We used STATA Version 13 (StataCorp) for data extraction and all the analyses including estimation for the national populations from samples and multivariable logistic regression. We provided the STATA commands for the year 2015 in the **Supplementary Materials**.

## Results

### Numbers of Patients With MDD and Their Antidepressant Treatment Over the Years

**Figure 1:**
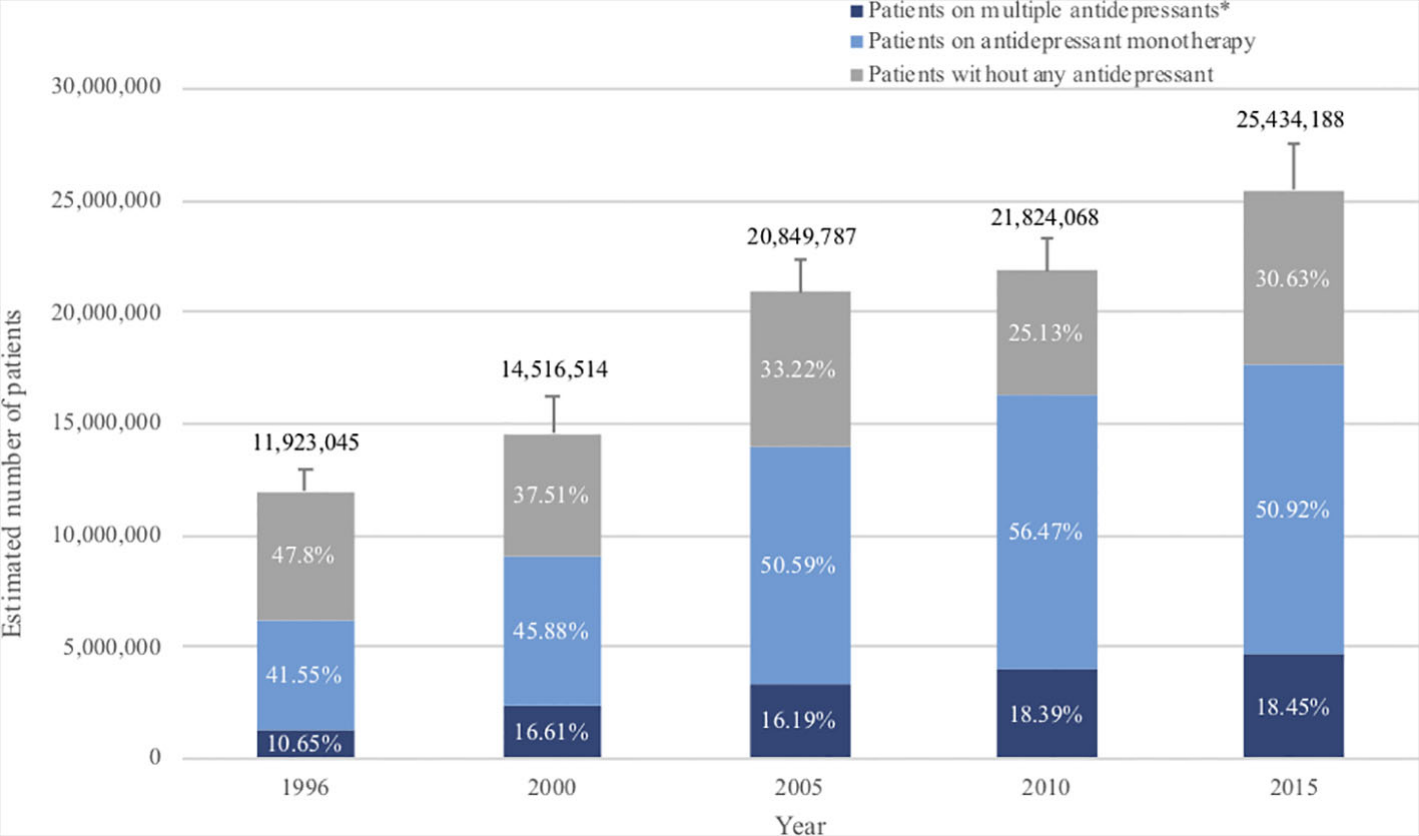
Antidepressant treatment for patients with major depression over the past 20 years. The standard error (SE) of number of adults with MDD is shown by the error bar. *Patients with multiple antidepressants: referring to patients who were prescribed with more than one antidepressant during that year, i.e. both patients with combination therapy and patients who changed previous monotherapy into a new drug in that year.

Estimated numbers of patients diagnosed with MDD showed constant increase (**Figure 1**). Prevalence of MDD among the adult population was 6.1% (95% CI, 5.7 to 6.6%) in 1996, which has increased steadily up to 2015, when it reached 10.4% (95% CI, 9.7 to 11.1%). Patients with a diagnosis of MDD who were not on any antidepressant treatment accounted for 47.8% (95% CI, 44.3 to 51.3%) of all patients in 1996, but the proportion gradually decreased to 25.1% (95% CI, 23.0 to 27.4%) in 2010 or 30.6% (95% CI, 28.3 to 33.1%) in 2015 (**Figure 1**).

### Characteristics of Patients With MDD Who Are on Antidepressant Monotherapy and Their Changes Over the Years

**Table 1** and **Supplementary Table S2** show characteristics of MDD patients who were prescribed with antidepressant monotherapy in the past 20 years. The mean age of these patients increased by about 10 years through the two decades, mainly due to the obvious increase of patients over 60 years. The sex ratio was roughly steady, with approximately 70% being women. The prescription numbers for male and female patients were shown separately in **Supplementary Figure S1**. The concomitant use of benzodiazepines was stable during the years at around 25%, whereas the use of mood stabilizers and antipsychotics increased from 3.1 to 5.6% and from 3.3 to 9.0%, respectively. More patients had long-term prescriptions of antidepressants in 2015, with 43.9% on antidepressants for more than 5 years, compared with only 13.4% in 1996. We further analyzed the proportion of long-term prescription of frequently prescribed drugs over the years (**Supplementary Figure S3**). In general, long-term prescription increased at approximately equal proportions for all the examined drugs. In 2015, for drugs known to cause discontinuation effect such as venlafaxine and paroxetine, almost 50% of their prescriptions (45.9 and 48.2%, respectively) were long-term uses. However, drugs less likely to cause withdrawal symptoms, for instance, fluoxetine, sertraline and bupropion, also had 46.4, 45.1 and 44.3% of prescriptions that have been used for more than 5 years respectively.

**Table 1:** Characteristics of depression patients on antidepressant monotherapy over the past 20 years.

Characteristics	1996 N = 4,954,122 n (%)	2000 N = 6,659,854 n (%)	2005 N = 10,548,016 n (%)	2010 N = 12,324,355 n (%)	2015 N = 12,950,609 n (%)
**Age, median (IQR), years**	46 (37, 59)	49 (39, 61)	50 (39, 61)	54 (42, 64)	56 (43, 66)
**Age group, years**					
18**–**29	506,770 (10.2)	600,663 (9.0)	1,155,148 (11.0)	1,231,686 (10.0)	1,099,211 (8.5)
30**–**39	953,467 (19.3)	1,130,306 (17.0)	1,501,298 (14.2)	1,560,833 (12.7)	1,602,932 (12.4)
40**–**49	1,320,960 (26.7)	1,725,914 (25.9)	2,404,993 (22.8)	2,084,911 (16.9)	1,976,450 (15.3)
50**–**59	972,364 (19.6)	1,395,414 (21.0)	2,484,401 (23.6)	3,205,742 (26.0)	2,968,142 (22.9)
≥60	1,200,562 (24.2)	1,807,557 (27.1)	3,002,176 (28.5)	4,241,182 (34.4)	5,303,873 (41.0)
**Sex**					
Male	1,427,443 (28.8)	1,619,797 (24.3)	3,300,937 (31.3)	3,978,123 (32.3)	4,067,234 (31.4)
Female	3,526,679 (71.2)	5,040,057 (75.7)	7,247,079 (68.7)	8,346,232 (67.7)	8,883,375 (68.6)
**Chronic physical conditions**^ab^	–	2,829,546 (43.7)	4,799,855 (45.8)	7,072,028 (57.4)	7,701,600 (59.7)
**PHQ-2 score (IQR)**^b^	–	–	2 (0, 3)	2 (0, 3)	1 (0, 2)
**PHQ-2 score**^b^	–	–			
**<** 3	–	–	6,961,550 (70.5)	8,529,753 (72.9)	8,933,938 (76.3)
≥3	–	–	2,910,081 (29.5)	3,171,618 (27.1)	2,775,204 (23.7)
**K6 score (IQR)**^b^	–	–	6 (3, 11)	6 (2, 11)	5 (2, 10)
**K6 score**^b^					
**<** 13	–	–	7,865,523 (79.8)	9,427,559 (80.7)	9,832,609 (86.3)
≥13	–	–	1,995,231 (20.2)	2,252,653 (19.3)	1,714,237 (13.7)
**Pain**^bc^					
Not at all	–	2,081,491 (33.0)	3,266,711 (32.5)	3,974,558 (33.6)	3,550,565 (29.8)
A little bit	–	1,742,303 (27.6)	2,591,903 (25.8)	3,336,528 (28.2)	3,280,701 (27.5)
Moderately	–	1,021,704 (16.2)	1,560,753 (15.5)	1,588,735 (13.4)	1,969,019 (16.5)
Quite a bit	–	974,881 (15.5)	1,859,447 (18.5)	1,812,975 (15.3)	2,243,139 (18.8)
Extremely	–	491,474 (7.8)	787,264 (7.8)	1,117,257 (9.5)	869,310 (7.3)
**Benzodiazepines**	1,240,483 (25.0)	1,198,566 (18.0)	2,500,583 (23.7)	3,098,868 (25.1)	3,437,484 (26.5)
**Mood stabilizers**	155,156 (3.1)	278,505 (4.2)	422,583 (4.0)	621,636 (5.0)	726, 451 (5.6)
**Antipsychotics**	163,569 (3.3)	258,977 (3.9)	679,955 (6.5)	1,009,160 (8.2)	1,166,052 (9.0)
**Duration of AD use, (IQR), years**^b^	1 (0, 2)	0 (0, 2)	1 (0, 3)	2 (0, 5)	3 (1, 10)
**Duration of AD use, years**^b^					
≤1	3,164,717 (65.6)	3,166,692 (72.0)	4,109,669 (61.0)	4,794,190 (43.6)	4,635,064 (39.1)
2**–**5	1,008,355 (20.9)	608,715 (13.8)	1,355,767 (20.1)	2,865,837 (26.0)	2,008,338 (17.0)
≥5	647,992 (13.4)	624,029 (14.2)	1,270,383 (9.3)	3,346,100 (30.4)	5,205,470 (43.9)
**Types of antidepressants**					
SSRIs	3,367,215 (68.0)	4,900,775 (73.6)	7,154,090 (67.8)	7,953,016 (64.5)	8,318,802 (64.2)
TCAs	870,096 (17.6)	704,692 (10.6)	603,409 (5.7)	396,197 (3.2)	418,713 (3.2)
SNRIs	165,336 (3.3)	348,478 (5.2)	1,396,424 (13.2)	2,024,762 (16.4)	2,118,382 (16.4)
Others	551,467 (11.1)	705,909 (10.6)	1,394,093 (13.2)	1,950,381 (15.8)	2,094,711 (16.2)

a Chronic physical conditions include any of the hypertension (HTN), coronary heart disease (CHD), stroke, diabetes mellitus (DM).

b Indicating that the variable had missing values. The missing value proportion in chronic physical conditions was 2.68%, 0.7% and 0.14% in 2000, 2005, 2015 respectively. The missing percentage in PHQ-2 scores was 6.41%, 5.05%, and 9.59% since 2005, while in K6 scores was 6.52%, 5.23, and 10.84%. Pain scores had a missing proportion at 5.23%, 4.57%, 4.01% and 8.02%. Duration of antidepressant use data were missing at the level of 2.69%, 33.94%, 36.14%, 10.7% and 8.51% respectively since 1996.

c Pain level was recorded according to one question from Short-Form 12 Version 2 (SF-12v2) that asked the participants the feeling of pain in the past 4 weeks.

IQR, interquartile range; PHQ, Patient Health Questionnaire; K6, Kessler Index; AD, antidepressant; TCA, tricyclic antidepressant; SSRI, serotonin reuptake inhibitor; SNRI, serotonin and norepinephrine reuptake inhibitor.

### Prescription Patterns of Antidepressant Monotherapy Among Patients With MDD Over the Years

**Figure 2:**
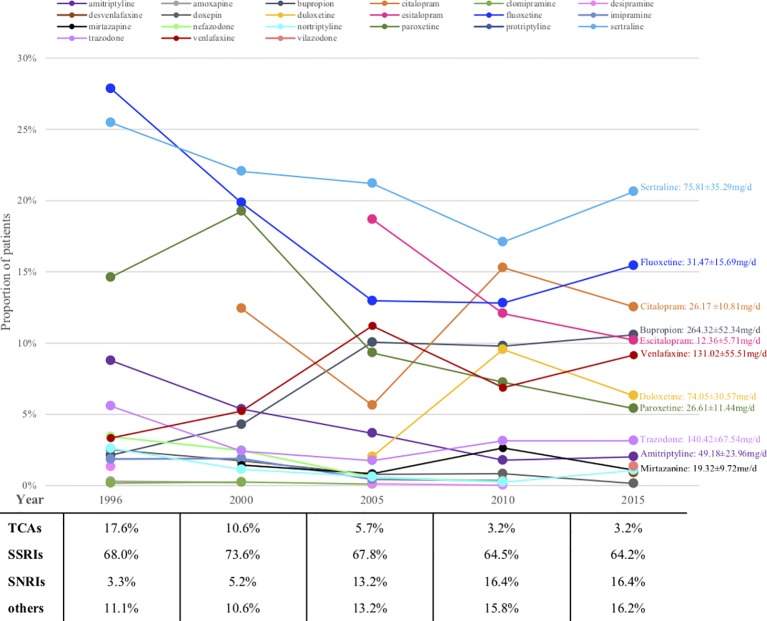
Prescriptions of antidepressants monotherapy for major depression patients over the years (proportions). TCA, tricyclic antidepressant; SSRI, serotonin reuptake inhibitor; SNRI, serotonin and norepinephrine reuptake inhibitor.

**Figure 2** shows the prescription patterns of individual antidepressants based on the proportion of patients being prescribed each drug among all patients on monotherapy. **Supplementary Table S3** shows the absolute numbers of patients on each drug estimated with 95% CI and **Supplementary Figure S2** depicts their trends over the years. Sertraline and fluoxetine were among the most prescribed antidepressants throughout the whole 20 years, with the absolute prescriptions increasing but prescription percentages decreasing, perhaps due to the introduction of more and more new drugs into the market in these years. Some relatively old antidepressants showed decrease both in absolute and relative numbers, such as paroxetine (from ranking the 3rd with 14.6% to ranking the 8th with 5.4%) and amitriptyline (from ranking the 4th with 8.8% to ranking the 10th with 2.0%), while some appeared be consistently prescribed although relatively infrequently (for example, trazodone). New drugs usually showed gradual increase, such as bupropion, venlafaxine and duloxetine, whereas several achieved surprisingly high prescription numbers upon their first appearance, such as escitalopram (dominating 18.7% and ranking the 2nd upon first appearance) and citalopram (occupying 12.4% and ranking the 4th upon first appearance).

Looking at antidepressant classes, SSRIs remained steady at around 70% for the whole 20 years, whereas TCAs were declining and SNRIs were growing all along (**Figure 2**).

### Average Dosages for Commonly Prescribed Antidepressant Monotherapy in 2015

**Figure 2** also shows the average daily dose of prescription for patients with MDD based on available data in 2015. The average doses of bupropion, trazodone and amitriptyline were lower than the therapeutic dose range approved by FDA.

### Factors Related to Suboptimal Prescriptions of Antidepressant Monotherapy in 2015

Data required for dose analysis were not complete in 43.1% of the major depressive patients on antidepressant monotherapy. Among the patients with sufficient data, 16.1% (95% CI, 12.5 to 20.2%) were prescribed with a dose lower than the approved range. After adjusting for age, sex and BMI, we discovered that patients being separated, widowed or divorced, or being prescribed with TCAs or any other antidepressants than SSRIs and SNRIs, tended to have higher risk to be prescribed with inadequate doses, while patients with higher BMI, or having long-term antidepressant treatment, had lower risk to receive inadequate prescriptions (**Table 2**). A multivariable regression implied that BMI, duration of antidepressant use and antidepressant type were the strongest factors related to suboptimal prescriptions (**Table 2**).

**Table 2:** Characteristics of patients prescribed with antidepressant monotherapy of suboptimal dose in 2015.

Characteristics	Patients on usual dosage (N = 6,186,819) n (%)	Patients on lower dosage (N = 1,179,536) n (%)	Patients on lower dosage, OR (95% CI)			
			Crude OR	Adjusted OR^a^	Multivariable OR^b^	*P* value for multivariable model
**Age group, years**						
18**–**29	7.5%	9.1%	Ref	Ref	Ref	
30**–**39	12.3%	13.0%	0.87 (0.28, 2.73)	1.14 (0.36, 3.57)	1.67 (0.43, 6.55)	0.457
40**–**49	15.0%	13.2%	0.72 (0.21, 2.42)	0.99 (0.30, 3.25)	1.60 (0.37, 6.93)	0.527
50**–**59	21.8%	24.3%	0.92 (0.30, 2.78)	1.39 (0.45, 4.30)	2.29 (0.59, 8.94)	0.232
≥60	43.5%	40.4%	0.76 (0.30, 1.94)	0.99 (0.40, 2.43)	1.80 (0.49, 6.67)	0.375
**Sex**						
Male	32.4%	25.3%	Ref	Ref	Ref	
Female	67.6%	74.7%	1.41 (0.81, 2.47)	1.40 (0.78, 2.51)	1.60 (0.72, 3.57)	0.249
**Race/ethnicity**						
White, non-Hispanic	83.5%	82.3%	Ref	Ref	Ref	
Black, non-Hispanic	5.3%	7.7%	1.46 (0.73, 2.93)	1.70 (0.81, 3.58)	1.81 (0.60, 5.42)	0.289
Hispanic	7.4%	8.6%	1.17 (0.61, 2.28)	1.17 (0.59, 2.33)	1.30 (0.51, 3.31)	0.576
Others	3.9%	1.5%	0.40 (0.11, 1.49)	0.41 (0.12, 1.44)	0.58 (0.18, 1.87)	0.360
**Education**^c^						
**<** High school graduate	11.8%	11.0%	Ref	Ref	Ref	
High school graduate	58.1%	61.5%	1.13 (0.65, 1.98)	1.08 (0.60, 1.95)	0.99 (0.42, 2.32)	0.984
College graduate	30.1%	27.5%	0.98 (0.48, 2.00)	0.96 (0.44, 2.09)	0.61 (0.18, 2.04)	0.419
**Marital status**						
Married	54.1%	40.0%	Ref	Ref	Ref	
Separated/divorced**/**widowed	29.5%	41.6%	1.91 (1.13, 3.22)	**1.97 (1.11, 3.48)**	1.98 (0.88, 4.46)	0.097
Not married	16.4%	18.4%	1.52 (0.73, 3.16)	1.28 (0.61, 2.72)	1.43 (0.55, 3.70)	0.462
**Family income level (%FPL)**						
**<** 100 (negative or poor)	14.1%	18.3%	Ref	Ref	Ref	
100**–**200 (low income)	18.3%	16.7%	0.70 (0.31, 1.59)	0.71 (0.31, 1.62)	0.49 (0.14, 1.80)	0.284
201**–**400 (middle)	31.3%	22.7%	0.56 (0.25, 1.24)	0.55 (0.25, 1.22)	0.45 (0.17, 1.15)	0.095
**>** 400 (high income)	36.4%	42.3%	0.89 (0.42, 1.88)	0.91 (0.42, 1.96)	0.93 (0.30, 2.86)	0.893
**Health insurance**						
None	3.4%	2.4%	0.72 (0.21, 2.42)	0.67 (0.19, 2.40)	0.83 (0.20, 3.49)	0.795
Public, only	30.8%	34.8%	1.18 (0.72, 1.93)	1.21 (0.72, 2.04)	0.86 (0.37, 1.97)	0.715
Private, any	65.7%	62.9%	Ref	Ref	Ref	
**PHQ-2 score (IQR)** ^c^	1 (0, 2)	1 (0, 2)	0.93 (0.83, 1.06)	0.94 (0.83, 1.07)	0.99 (0.75, 1.31)	0.961
**K6 score (IQR)** ^c^	5 (2, 10)	5 (2, 9)	0.99 (0.95, 1.04)	0.99 (0.95, 1.04)	0.96 (0.87, 1.05)	0.354
**Chronic physical conditions**^d^	61.2%	55.0%	0.77 (0.48, 1.23)	1.04 (0.60, 1.79)	0.84 (0.41, 1.69)	0.615
**Cancer**	16.0%	20.1%	1.32 (0.66, 2.63)	1.53 (0.76, 3.05)	1.54 (0.68, 3.47)	0.275
**BMI, median (IQR), kg/m^2^** ^c^	29.5 (25, 35.2)	26.4 (23, 31.2)	0.94 (0.90, 0.98)	**0.94 (0.90, 0.98)**	**0.94 (0.90, 0.99)**	***0.017***
**Obvious pain**^ce^	22.8%	16.6%	0.63 (0.35, 1.13)	0.67 (0.35, 1.26)	0.65 (0.26, 1.62)	0.349
**Duration of AD use, (IQR), years**^c^	4 (1, 11)	2 (0, 5)	0.93 (0.88, 0.99)	**0.93 (0.88, 0.99)**	**0.92 (0.87, 0.97)**	***0.003***
**Type of antidepressants**						
SSRIs	67.2%	51.7%	Ref	Ref	Ref	
TCAs	2.0%	9.2%	6.04 (2.01, 18.13)	**6.61 (2.09, 20.86)**	**11.21 (2.12, 59.34)**	***0.005***
SNRIs	18.1%	8.8%	0.63 (0.28, 1.43)	0.59 (0.26, 1.33)	0.81 (0.35, 1.87)	0.621
Others	12.8%	30.4%	3.10 (1.71, 5.63)	**3.35 (1.76, 6.36)**	**4.12 (1.95, 8.73)**	***<0.001***

a Adjusted by age, sex and BMI.

b Multivariable regression model included independent variables of age, sex, race, education, marital status, family income level, health insurance, BMI, chronic diseases, cancer, obvious pain, duration of antidepressant use, PHQ-2 scores, K6 scores, and type of antidepressants.

c Indicating that the variable had missing values. The missing value proportion in education was 0.63% and 0.84% in the group of usual dose and lower dose respectively. The missing proportion in K6 score was 11.29% and 8.61% in 2 groups while in PHQ-2 was 10.47% and 6.42% respectively. The missing percentage in BMI was 2.03% in the usual dose group. Pain data had a missing report of 8.67% and 3.93% in the usual dose and lower dose group. Duration of antidepressant use data were missing at the level of 7.63% in the usual dose group, and 8.41% in the lower dose group respectively.

d Chronic physical conditions include any of the hypertension (HTN), coronary heart disease (CHD), stroke, diabetes mellitus (DM).

e Obvious pain was defined as a pain evaluated as “quite a bit” or “extremely”.

OR, odds ratio; IQR, interquartile range; FPL, federal poverty level; PHQ, Patient Health Questionnaire; K6, Kessler Index; BMI, body mass index; AD, antidepressant; TCA, tricyclic antidepressant; SSRI, serotonin reuptake inhibitor; SNRI, serotonin and norepinephrine reuptake inhibitor. The p-values for the bolded text are: Marital status: Separated/divorced/widowed/not married 1.97 (1.11, 3.48), p = 0.021; BMI: 0.94 (0.90, 0.98), p = 0.003; Duration of AD use: 0.93 (0.88, 0.99), p = 0.021; Type of antidepressants: TCAs: 6.61 (2.09, 20.86), p = 0.001; Others: 3.35 (1.76, 6.36), p < 0.001.

## Discussion

We found that the absolute and relative numbers of adult patients diagnosed with MDD increased over the past 20 years, as well as the proportion of those on antidepressant treatment among those so diagnosed. There were approximately 30% of such patients who were not on any antidepressants in 2015. Among those who were on antidepressant monotherapy, there was substantial increase in long-term prescriptions and some increase in concurrent use of mood stabilizer or antipsychotics. The prescription patterns of specific drugs changed over the years as new antidepressants came into the market continuously. Sertraline and fluoxetine were among the most frequently prescribed antidepressants throughout these 20 years, while new drugs such as citalopram and escitalopram were prescribed by a dramatically large amount soon after their entry into the market. On the other hand, 16.1% of patients were using antidepressants below the licensed doses, especially when the patients had lower BMI, had shorter length of treatment, and were prescribed antidepressants other than SSRIs and SNRIs.

The prevalence of adult major depression estimated in our study was between 6.1 and 10.4% from 1996 to 2015, which was in line with the epidemiological studies from the same periods (20–23). The constantly growing total number of patients with MDD calls for more attention on how to implement effective interventions and care for the patients.

Antidepressants are one of the principal treatments for MDD, but still quite a few were not prescribed with any antidepressants. An antidepressant was originally recommended as the initial treatment for patients with moderate to severe depression by several guidelines (8, 11, 12, 24), whereas APA guideline recommends antidepressant as the first-line treatment also for mild patients (10, 25). Two individual participant data meta-analysis (26, 27) indicated that patients with lower baseline severity would achieve smaller improvement compared to placebo. However, a more recent study (28) found that the differential response of patients with different severity was due to larger improvement on non-core symptoms, and that baseline severity did not affect the efficacy for core depression symptoms. Besides, as we did not have the data for baseline severity or the treatment course for individual patients, we could not judge appropriateness of prescribing or not prescribing an antidepressant in individual cases or further explore the factors related to not receiving antidepressant treatment.

Our data suggested that there was dramatic increase in long-term prescriptions of antidepressant monotherapy, which was also observed by some other studies (29, 30). This phenomenon might be due to the increased prescriptions as appropriate maintenance treatment for patients with recurrent episodes, or due to improperly elongated use related to withdrawal symptoms, or both. Our results suggested that frequently prescribed drugs tended to have large proportion of prescriptions to be long-term, apparently regardless of the risk to cause withdrawal symptoms. It may imply that discontinuation syndrome might not be the only reason that caused significant increase in long-term prescriptions. The current observational study could not provide any further conclusions for this phenomenon, thus future studies are required. Although long-term maintenance treatment is recommended for patients with recurrent episodes (10, 12), future studies are required to explore the appropriateness of actual prolonged prescriptions (31, 32).

In the US an old study (33) based on office-based physician survey depicted the trend of antidepressant prescriptions for depression up to 2001, by which time SSRIs had clearly outnumbered TCAs. In most countries in Europe, SSRIs were the class being prescribed most frequently in 2004–2005, especially in France and the UK, whereas in Germany TCAs dominated (34). In Asia, though SSRIs dominated in almost every country, the particular prescription preferences were different from country to country (35–37). These various prescription patterns might be attributable to the perception that no single antidepressant appears much better than another, which in turn might suggest that particular marketing conditions and regulations, adverse effect spectrum and patients' preferences might impact greatly on the actual prescription patterns. As evidence accrues, we need to rigorously summarize it which then should guide us in actual prescriptions and should no longer let individual experiences or marketing efforts to distort it.

Several studies have pointed to the suboptimal prescription of antidepressants. A few studies suggested that older antidepressants such as TCAs were more susceptible to be prescribed in low doses (34, 38, 39), which was consistent with our study. Some studies further revealed that low dose prescriptions were especially related to primary care physicians, perhaps due to their concerns about the side effects related to TCAs, or the lack of confidence of those general practitioners (34, 38, 40). Besides, some antidepressants might be prescribed for their hypnotic effect rather than for depressive symptoms, such as amitriptyline or trazodone, which could lead to prescriptions with smaller doses. In our study, patients with longer-term prescription were less likely to receive inadequate doses, which might be ascribed to the fact that most long-term users were clinically severe or refractory so that sufficient doses were indispensable. Lower BMI was also associated of suboptimal prescription. This may be clinically understandable, because patients with less body weight may need lower dose, or they may be more likely to show adverse effects. The clinicians may also take advantage of placebo effect when it presents before the licensed dose range is achieved.

This study has some limitations. First, prescription of antidepressants is actually different from their real consumption. Although the MEPS is a large survey database with rigorous methodology based on representative samples in the US, some important information was not recorded, such as depression severity or treatment responses, the specialty of the doctor who prescribers a certain drug, among others. Second, even when recorded, some variables such as quantity of prescribed medications were often missing, thus prohibiting the calculation of average daily doses for some patients. Also, some of the available information was not very precise. For instance, the diagnosis in the household component of the MEPS is mainly dependent on patients' report. Although the sensitivity of a broader diagnosis in mental health disorders reported is above 90%, the specific diagnosis might be less uncertain, as addressed in a case study (41). However, there are several previous studies using the MEPS and their results are comparable with results generated from other sources (42–44). In addition, the combination antidepressant treatment was difficult to define. It was also not possible to distinguish incident patients from chronic patients therefore both acute phase and maintenance treatment were included in our analyses. These limitations may make some inferential statements of the observations challenging, and future studies focusing on these points are warranted.

In the literature, most population-based prescription studies are based on claims databases where the diagnoses were uncertain, while small cohort studies of patients with established diagnoses were usually institution-based with short-term follow-up and therefore had problems in generalizability. Our study represents the first detailed descriptions of population-based, long-term trends of antidepressant prescription patterns for patients diagnosed with MDD in the US. It has once again pointed to the increasing numbers of patients with MDD and also the increase in the antidepressant prescriptions among them. At the same time, it has revealed some unresolved gaps between evidence and practice, most notably existence of substantial minorities without any antidepressant prescriptions or with only subtherapeutic prescriptions among those diagnosed with MDD, dramatic increase in the number of patients with extremely long-term antidepressant prescriptions, and variable patterns in choices of individual antidepressants. These gaps need be filled in by independently funded future research.

## Data Availability Statement

The datasets used in this study are publicly available in the Medical Expenditure Panel Survey (MEPS), https://meps.ahrq.gov/mepsweb/.

## Ethics Statement

Ethical approval was not required as publicly available datasets were analysed.

## Author Contributions

YL and TF designed the study. YL collected data and conducted statistical analyses. TF, YK, and EO gave suggestions for analytical plans. All the authors participated in interpretation of the results. YL drafted the manuscript and all authors critically revised the manuscript and approved the final version.

## Funding

This study was supported in part by JSPS Grant-in-Aid for Scientific Research (Grant Number 17k19808) to TF. The funder has no role in study design, data collection, data analysis, data interpretation, writing of the report, or in the decision to submit for publication.

## Conflict of Interest

TF reports personal fees from Meiji, Mitsubishi-Tanabe, MSD and Pfizer and a grant from Mitsubishi-Tanabe, outside the submitted work; TF has a patent 2018-177688. AC is supported by the National Institute for Health Research (NIHR) Oxford Cognitive Health Clinical Research Facility, by an NIHR Research Professorship (grant RP-2017-08-ST2-006) and by the NIHR Oxford Health Biomedical Research Centre (grant BRC-1215-20005). The views expressed are those of the authors and not necessarily those of the UK National Health Service, the NIHR, or the UK Department of Health.

The remaining authors declare that the research was conducted in the absence of any commercial or financial relationships that could be construed as a potential conflict of interest.
