# Corrigendum: National Prescription Patterns of Antidepressants in the Treatment of Adults With Major Depression in the US Between 1996 and 2015: A Population Representative Survey Based Analysis

**DOI:** 10.3389/fpsyt.2020.00171

**Published:** 2020-03-06

**Authors:** Yan Luo, Yuki Kataoka, Edoardo G. Ostinelli, Andrea Cipriani, Toshi A. Furukawa

**Affiliations:** ^1^Department of Health Promotion and Human Behavior, School of Public Health in the Graduate School of Medicine, Kyoto University, Kyoto, Japan; ^2^Hospital Care Research Unit, Hyogo Prefectural Amagasaki General Medical Center, Hyogo, Japan; ^3^Department of Health Sciences, Università degli Studi di Milano, Milan, Italy; ^4^Department of Psychiatry, Warneford Hospital, University of Oxford, Oxford, United Kingdom

**Keywords:** major depressive disorder, antidepressant, prescription, trend, suboptimal dose

In the original article the CFACT Data Center and AHRQ were not acknowledged. The updated **Acknowledgments** section appears below.

“The research in this paper was conducted at the CFACT Data Center, and the support of AHRQ is acknowledged. The results and conclusions in this paper are those of the authors and do not indicate concurrence by AHRQ or the Department of Health and Human Services. We would like to thank all the members from the meta-epidemiological study group in the School of Public Health, Kyoto University, for the constructive comments and advice.”

The authors apologize for this error and state that this does not change the scientific conclusions of the article in any way. The original article has been updated.

