# Supplementary material for: National Prescription Patterns of Antidepressants in the Treatment of Adults With Major Depression in the US Between 1996 and 2015: A Population Representative Survey Based Analysis
Source: Front Psychiatry. 2020 Feb 14;11:35. doi: 10.3389/fpsyt.2020.00035 (PMC7033625; doi:10.3389/fpsyt.2020.00035)
Supplement: Supplementary file 1 [file DataSheet_1.docx]

# SUPPLEMENTARY MATERIALS

## Tables

**Table S1. Therapeutic dose ranges approved by Food and Drug Administration (FDA) (mg/day) for adults of depression**

| Antidepressant^a^ | Therapeutic dose range (mg/d)^b^ |
| --- | --- |
| Amitriptyline | 50-200 |
| Bupropion | 300-450 |
| Citalopram | 20-40 |
| Clomipramine | 30-250 |
| Desipramine | 100-300 |
| Desvenlafaxine | 50-100 |
| Duloxetine | 40-120 |
| Escitalopram | 10-20 |
| Fluoxetine | 20-80 |
| Imipramine | 75-300 |
| Levomilnacipran | 40-120 |
| Mirtazapine | 15-45 |
| Nefazodone | 300-600 |
| Nortriptyline | 75-150 |
| Paroxetine | 20-50 |
| Protriptyline | 15-60 |
| Sertraline | 50-200 |
| Trazodone | 150-400 |
| venlafaxine | 75-375 |
| Vilazodone | 20-40 |
| Vortioxetine | 5-20 |

*^a^Only those being prescribed in 2015 in MEPS were included.*

*^b^Starting dose was excluded if it was supposed to be increased. Doses recommended for the adolescents, the elderly, or other particular patient groups were not taken into account.*

**Table S2. Other sociodemographic characteristics of depression patients on antidepressant monotherapy over the past 20 years**

| Characteristics | 1996  N=4,954,122  n (%) | 2000  N=6,659,854  n (%) | 2005  N=10,548,016  n (%) | 2010  N=12,324,355  n (%) | 2015  N=12,950,609  n (%) |
| --- | --- | --- | --- | --- | --- |
| Region |  |  |  |  |  |
| Northeast | 980,819 (19.8) | 1,305,181 (19.6) | 1,768,903 (16.77) | 2,182,832 (17.71) | 2,436,060 (18.81) |
| Midwest | 1,351,459 (27.28) | 1,434,584 (21.54) | 2,706,353 (25.66) | 3,182,012 (25.82) | 3,219,061 (24.86) |
| South | 1,643,377 (33.17) | 2,513,211 (37.74) | 3,750,525 (35.56) | 4,453,245 (36.13) | 4,666,376 (36.03) |
| West | 978,467 (19.75) | 1,406,878 (21.12) | 2,322,235 (22.02) | 2,506,266 (20.34) | 2,629,111 (20.30) |
| Race/ethnicity |  |  |  |  |  |
| White, non-Hispanic | 4,204,344 (84.87) | 5,851,470 (87.86) | 8,807,464 (83.5) | 10,303,701 (83.6) | 10,532,035 (81.32) |
| Black, non-Hispanic | 345,345 (6.97) | 232,452 (3.49) | 613,894 (5.82) | 619,756 (5.03) | 772,433 (5.96) |
| Hispanic | 331,799 (6.7) | 455,808 (6.84) | 760,219 (7.21) | 877,828 (7.12) | 1,137,715 (8.79) |
| Others | 72,635 (1.47) | 120,125 (1.8) | 366,439 (3.47) | 523,070 (4.24) | 508,426 (3.93) |
| Education^a^ |  |  |  |  |  |
| < High school graduate | 1,197,095 (24.16) | 1,439,904 (21.79) | 2,076,346 (19.74) | 2,141,462 (17.42) | 1,439,692 (11.18) |
| High school graduate | 2,587,628 (52.23) | 3,219,601 (48.72) | 5,721,411 (54.41) | 6,686,930 (54.40) | 7,676,456 (59.60) |
| College graduate | 1,169,399 (23.6) | 1,948,955 (29.49) | 2,717,006 (25.84) | 3,465,237 (28.18) | 3,762,667 (29.21) |
| Marital status |  |  |  |  |  |
| Married | 2,450,643 (49.47) | 3,204,444 (48.12) | 5,676,935 (53.82) | 6,304,216 (51.15) | 6,573,758 (50.76) |
| Separated/divorced/ widowed | 1,589,424 (32.08) | 2,328,786 (34.97) | 3,113,295 (29.52) | 3,784,975 (30.71) | 4,232,053 (32.68) |
| Not married | 914,056 (18.45) | 1,126,624 (16.92) | 1,757,787 (16.66) | 2,235,164 (18.14) | 2,144,797 (16.56) |
| Family total income, median (IQR), dollars | - | - | - | 47200 (22000, 80000) | 49905 (21840, 87089) |
| Family income level (%FPL) |  |  |  |  |  |
| <100 (negative or poor) | 784,482 (15.83) | 859,423 (12.9) | 1,395,584 (13.23) | 1,785,456 (14.49) | 2,051,872 (15.84) |
| 100-200 (low income) | 962,419 (19.43) | 1,318,672 (19.8) | 1,882,289 (17.84) | 2,302,817 (18.69) | 2,350,833 (18.15) |
| 201-400 (middle) | 1,596,567 (32.23) | 2,220,898 (33.35) | 3,277,036 (31.07) | 3,547,077 (28.78) | 3,750,823 (28.96) |
| >400 (high income) | 1,610,654 (32.51) | 2,260,860 (33.95) | 3,993,108 (37.86) | 4,689,006 (38.05) | 4,797,081 (37.04) |
| Health insurance |  |  |  |  |  |
| None | 230,475 (4.65) | 493,575 (7.41) | 712,339 (6.75) | 737,405 (5.98) | 391,624 (3.02) |
| Public, only | 898,117 (18.13) | 1,514,984 (22.75) | 2,490,814 (23.61) | 3,427,433 (27.81) | 4,065,095 (31.39) |
| Private, any | 3,825,531 (77.22) | 4,651,295 (69.84) | 7,344,863 (69.63) | 8,159,516 (66.21) | 8,493,890 (65.59) |
| BMI, median (IQR), kg/m^2 a^ | - | - | 27.5 (24, 32.6) | 28.8 (24.7, 34.2) | 28.9 (24.4, 34.4) |
| BMI group, kg/m^2 a^ |  |  |  |  |  |
| < 18.5 | - | - | 162,007 (1.58) | 133,056 (1.10) | 200,280 (1.58) |
| 18.5-24.9 | - | - | 3,163,118 (30.84) | 3,003.623 (24.84) | 3,289,556 (25.89) |
| 25-29.9 | - | - | 3,091,030 (30.13) | 3,824,267 (31.62) | 3,537,142 (27.84) |
| ≥ 30 | - | - | 3,839,275 (37.44) | 5,132,049 (42.44) | 5,679,003 (44.69) |

**^a^**The variable had missing values. The missing value proportion in education was 0.77%, 0.32%, 0.25% and 0.55% in 2000, 2005, 2010, 2015 respectively. The missing percentage in BMI was 2.77%, 1.88%, and 1.89% since 2005.

Abbreviations: IQR, interquartile range; FPL, federal poverty level; BMI, body mass index.

**Table S3. Absolute numbers of prescriptions of antidepressants monotherapy**

**for patients of MDD over the years**

| Drug name | 1996  N, 95%CI | 2000  N, 95%CI | 2005  N, 95%CI | 2010  N, 95%CI | 2015  N, 95%CI |
| --- | --- | --- | --- | --- | --- |
| Amitriptyline | 434,940  (279,257 to 590,623) | 357,705  (204,096 to 511,315) | 387,427  (225,844 to 549,010) | 219,697  (132,082 to 307,315) | 261,951  (151,548 to 372,354) |
| Amoxapine | 14,739  (-5,919 to 35,396) | 13,881  (-13,502 to 41,264) | 0 | 0 | 0 |
| Bupropion | 105,404  (19,997 to 190,811) | 285,156  (141,184 to 429,127) | 1,060,378  (8,023,33 to 1,318,423) | 1,206,859  (875,565 to 1,538,153) | 1,370,152  (1,049,794 to 1,690,509) |
| Citalopram | 0 | 827,878  (548,013 to 1,107,742) | 594,963  (421,545 to 768,380) | 1,885,171  (1,522,691 to 2,247,651) | 1,624,749  (1,264,650 to 1,984,848) |
| Clomipramine | 8,205  (-7,910 to 24,316) | 16,714  (-16,257 to 49,686) | 10,396  (-10,083 to 30,876) | 0 | 0 |
| Desipramine | 65,802  (12,144 to 119,459) | 0 | 12,771  (-6,447 to 31,988) | 2,612  (-2,538 to 7,762) | 0 |
| Desvenlafaxine | 0 | 0 | 0 | 0 | 116,895  (53,889 to 179,901) |
| Doxepin | 124,977  (48,454 to 201,500) | 115,335  (41,435 to 189,235) | 83,340  (26,420 to 140,261) | 102,313  (20,543 to 184,083) | 18,496  (-3,342 to 40,335) |
| Duloxetine | 0 | 0 | 214,630  (115,855 to 313,405) | 1,177,634  (863,967 to 1,491,300) | 816,686  (585,670 to 1,047,703) |
| Esitalopram | 0 | 0 | 1,970,846  (1,620,995 to 2,320,697) | 1,488,741  (1,088,576 to 1,888,906) | 1,322,050  (1,003,499 to 1,640,600) |
| Fluoxetine | 1,380,602  (1,084,663 to 1,676,541) | 1,322,226  (1,007,461 to 1,636,991) | 1,367,586  (1,086,069 to 1,649,103) | 1,579,148  (1,280,577 to 1,877,718) | 2,000,340  (1,599,398 to 2,401,281) |
| Imipramine | 92,279  (24,792 to 159,766) | 126,221  (34,829 to 217,614) | 45,082  (-7,680 to 97,844) | 43,294  (-11,269 to 97,857) | 0 |
| Mirtazapine | 0 | 95,240  (25,385 to 165,094) | 87,627  (31,232 to 144,022) | 325,121  (147,633 to 502,610) | 141,770  (57,485 to 226,055) |
| Nefazodone | 169,823  (79,200 to 260,446) | 164,373  (72,344 to 256,401) | 61,749  (8,847 to 114,650) | 32,678  (-14,235 to 79,591) | 0 |
| Nortriptyline | 129,115  (38,587 to 219,723) | 74,835  (11,519 to 138,151) | 64,393  (10,209 to 118,578) | 28,281  (-12,640 to 69,201) | 138,265  (42,778 to 233,753) |
| Paroxetine | 724,503  (492,135 to 956,871) | 1,281,585  (1,050,481 to 1,512,689) | 783,406  (758,132 to 1,208,681) | 893,447  (640,636 to 1,146,258) | 700,121  (495,682 to 904,560) |
| Protriptyline | 0 | 0 | 0 | 0 | 0 |
| Sertraline | 1,262,110  (1,011,188 to 1,513,033) | 1,469,087  (1,143,829 to 1,794,345) | 2,237,288  (1,8646,277 to 2,610,299) | 2,106,509  (1,716,035 to 2,496,983) | 2,671,543  (2,172,920 to 2,172,920) |
| Trazodone | 276,249  (137,992 to 414,505) | 161,141  (50,647 to 271,635) | 184,339  (82,627 to 286,052) | 385,722  (216,013 to 555,432) | 406,933  (225,488 to 588,378) |
| Venlafaxine | 165,336  (73,971 to 256,701) | 348,478  (202,256 to 494,700) | 1,181,794  (928,894 to 1,434,694) | 847,128  (574,344 to 1,119,912) | 1,184,801  (918,864 to 1,450,738) |
| Vilazodone | 0 | 0 | 0 | 0 | 175,857  (67,433 to 284,281) |
| Total | 4,954,122  (4,402,425 to 5,505,820) | 6,659,854  (5,841,641 to 7,478,067) | 10,548,016  (9,572,500 to 11,523,533) | 12,324,355  (11,280,432 to 13,368,279) | 12,950,609  (11,686,914 to 14,214,303) |

## Figures

**Figure S1. Antidepressant monotherapy prescriptions in male and female major depression patients**

**Figure S2. Prescriptions of antidepressants monotherapy for major depression patients over the years** (absolute numbers)

**Figure S3. Proportion of long-term prescriptions for frequently prescribed antidepressants over time**

## STATA commands (in 2015)

use "medical condition dta in 2015"

keep dupersid icd9code

gen depression=0

replace depression=1 if strmatch(icd9code, "296.2*") | strmatch(icd9code, "296.3*") | strmatch(icd9code, "311*")

drop if depression==0

duplicates drop dupersid, force

save diagnosis2015.dta, replace //patient ID with depression

clear

use "population characteristics dta in 2015"

keep dupersid age15x sex racethx edrecode marry15x faminc15 povcat15 inscov15 phq242 adpain42 region15 hibpdx chddx strkdx cancerdx diabdx arthdx bmindx53 pcs42 mcs42 k6sum42 perwt15f varstr varpsu

merge 1:m dupersid using diagnosis2015.dta

replace depression=0 if depression==.

save demographic_dignosis2015.dta, replace //diagnosis information merged in demographic file

clear

use "prescription dta in 2015"

keep dupersid linkidx rxdrgnam

duplicates drop linkidx, force

//unite the drug name & focus on ADs/benzo/mood stabilizers/antipsychotics

gen drug="others"

replace drug="amit" if strmatch(rxdrgnam,"AMITR*") | strmatch(rxdrgnam,"AMITID*") | strmatch(rxdrgnam,"ELAVIL*") | strmatch(rxdrgnam,"ENDEP")

replace drug="amox" if strmatch(rxdrgnam,"AMOXA*") | strmatch(rxdrgnam,"ASENDIN*")

replace drug="bupr" if strmatch(rxdrgnam,"BUPROP*") | strmatch(rxdrgnam,"APLENZIN*") | strmatch(rxdrgnam,"FORFIVO*") | strmatch(rxdrgnam,"WELLBUTRIN*") | strmatch(rxdrgnam,"ZYBAN") | strmatch(rxdrgnam,"BUPROBAN") | strmatch(rxdrgnam,"BUDEPRON")

replace drug="cita" if strmatch(rxdrgnam,"CITALO*") | strmatch(rxdrgnam,"CELEXA*")

replace drug="clom" if strmatch(rxdrgnam,"CLOMIPRAM*") | strmatch(rxdrgnam,"ANAFRANIL*")

replace drug="desi" if strmatch(rxdrgnam,"DESIPRAM*") | strmatch(rxdrgnam,"NORPRAMIN*") | strmatch(rxdrgnam,"PERTOFRANE*")

replace drug="desv" if strmatch(rxdrgnam,"DESVENLA*") | strmatch(rxdrgnam,"KHEDEZLA*") | strmatch(rxdrgnam,"PRISTQ*")

replace drug="doxe" if strmatch(rxdrgnam,"DOXEPIN*") | strmatch(rxdrgnam,"SILENOR*") | strmatch(rxdrgnam,"SINEQUAN*") | strmatch(rxdrgnam,"ZONALON*")

replace drug="dulo" if strmatch(rxdrgnam,"DULOXE*") | strmatch(rxdrgnam,"CYMBALTA*")

replace drug="esci" if strmatch(rxdrgnam,"ESCITALOP*") | strmatch(rxdrgnam,"LEXAPRO*")

replace drug="fluo" if strmatch(rxdrgnam,"FLUOXE*") | strmatch(rxdrgnam,"FLUOEXT*") | strmatch(rxdrgnam,"PROZAC*") | strmatch(rxdrgnam,"PROSAC") | strmatch(rxdrgnam,"SARAFEM*") | strmatch(rxdrgnam,"SELFEMRA*")

replace drug="imip" if strmatch(rxdrgnam,"IMIPRAM*") | strmatch(rxdrgnam,"PRAMINE*") | strmatch(rxdrgnam,"PRESAMINE*") | strmatch(rxdrgnam,"TOFRANIL*")

replace drug="levo" if strmatch(rxdrgnam,"LEVOMILNACIP*") | strmatch(rxdrgnam,"FETZIMA*")

replace drug="mirt" if strmatch(rxdrgnam,"MIRTAZ*") | strmatch(rxdrgnam,"REMERON*")

replace drug="nefa" if strmatch(rxdrgnam,"NEFAZO*") | strmatch(rxdrgnam,"SERZONE*")

replace drug="nort" if strmatch(rxdrgnam,"NORTRIP*") | strmatch(rxdrgnam,"AVENTYL*") | strmatch(rxdrgnam,"PAMELOR*")

replace drug="paro" if strmatch(rxdrgnam,"PAROXE*") | strmatch(rxdrgnam,"BRISDELLE*") | strmatch(rxdrgnam,"PAXIL*") | strmatch(rxdrgnam,"PEXEVA*")

replace drug="prot" if strmatch(rxdrgnam,"PROTRIP*") | strmatch(rxdrgnam,"VIVACTIL*")

replace drug="sert" if strmatch(rxdrgnam,"SERTR*") | strmatch(rxdrgnam,"ZOLOFT*")

replace drug="traz" if strmatch(rxdrgnam,"TRAZO*") | strmatch(rxdrgnam,"TRAZAD*") | strmatch(rxdrgnam,"DESYREL*") | strmatch(rxdrgnam,"TRIALODIN*")

replace drug="trim" if strmatch(rxdrgnam,"TRIMIPRAM*") | strmatch(rxdrgnam,"SURMONTIL*")

replace drug="venl" if strmatch(rxdrgnam,"VENLAF*") | strmatch(rxdrgnam,"EFFEXOR*")

replace drug="vila" if strmatch(rxdrgnam,"VILAZOD*") | strmatch(rxdrgnam,"VIIBRYD*")

replace drug="vort" if strmatch(rxdrgnam,"VORTIOX*")

replace drug="benzo" if strmatch(rxdrgnam,"BENZODIAZ*") | strmatch(rxdrgnam,"ALPRAZ*") | strmatch(rxdrgnam,"NIRAVAM*") | strmatch(rxdrgnam,"XANAX*") | strmatch(rxdrgnam,"CHLORDIA*") | strmatch(rxdrgnam,"A-POXIDE*") | strmatch(rxdrgnam,"LIBR*") | strmatch(rxdrgnam,"LIMBI*") | strmatch(rxdrgnam,"LYGEN") | strmatch(rxdrgnam,"MENRIUM*") | strmatch(rxdrgnam,"CLOBA*") | strmatch(rxdrgnam,"ONFI") | strmatch(rxdrgnam,"SIMPAZ*") | strmatch(rxdrgnam,"CLONAZEPAM") | strmatch(rxdrgnam,"KLONOPIN*") | strmatch(rxdrgnam,"CLORAZEP*") | strmatch(rxdrgnam,"GEN-XENE") | strmatch(rxdrgnam,"TRANXENE") | strmatch(rxdrgnam,"DIAZEPAM*") | strmatch(rxdrgnam,"DIASTAT*") | strmatch(rxdrgnam,"DIZAC") | strmatch(rxdrgnam,"Q-PAM") | strmatch(rxdrgnam,"VALIUM*") | strmatch(rxdrgnam,"VALRELEASE*") | strmatch(rxdrgnam,"ESTAZOLAM*") | strmatch(rxdrgnam,"PROSOM") strmatch(rxdrgnam,"FLURAZEPAM*") | strmatch(rxdrgnam,"DALMANE*") | strmatch(rxdrgnam,"HALAZEPAM*") | strmatch(rxdrgnam,"PAXIPAM*") | strmatch(rxdrgnam,"LORAZ*") | strmatch(rxdrgnam,"ATIVAN") | strmatch(rxdrgnam,"MIDAZOLAM*") | strmatch(rxdrgnam,"SEIZALAM*") | strmatch(rxdrgnam,"VERSED") | strmatch(rxdrgnam,"OXAZEPAM*") | strmatch(rxdrgnam,"SERAX")| strmatch(rxdrgnam,"ZAXOPAM*") | strmatch(rxdrgnam,"QUAZEPAM*") | strmatch(rxdrgnam,"DORAL*") | strmatch(rxdrgnam,"TEMAZ*") | strmatch(rxdrgnam,"RESTORIL*") | strmatch(rxdrgnam,"TRIAZOLAM*") | strmatch(rxdrgnam,"HALCION*") | strmatch(rxdrgnam,"FLUMAZEN*") | strmatch(rxdrgnam,"ROMAZI") | strmatch(rxdrgnam,"ESZOPI*") | strmatch(rxdrgnam,"LUNESTA*") | strmatch(rxdrgnam,"ZALEPLON*") | strmatch(rxdrgnam,"SONATA*") | strmatch(rxdrgnam,"ZOLPI*") | strmatch(rxdrgnam,"AMBIEN") | strmatch(rxdrgnam,"EDLUAR*") | strmatch(rxdrgnam,"INTERMEZ*") | strmatch(rxdrgnam,"TOVALT*")

replace drug="moodstabilizer" if strmatch(rxdrgnam,"*CARBAMAZ*") | strmatch(rxdrgnam,"CARBATROL*") | strmatch(rxdrgnam,"CARNEXIV*") | strmatch(rxdrgnam,"EPITOL*") | strmatch(rxdrgnam,"EQUETRO*") | strmatch(rxdrgnam,"TERIL") | strmatch(rxdrgnam,"TEGRETOL*") | strmatch(rxdrgnam,"VALPRO*") | strmatch(rxdrgnam,"DEPACON*") | strmatch(rxdrgnam,"DEPAK*") | strmatch(rxdrgnam,"STAVZOR*") | strmatch(rxdrgnam,"DIVALP*") | strmatch(rxdrgnam,"LITHIU*") | strmatch(rxdrgnam,"LITHANE*") | strmatch(rxdrgnam,"LITHOB*") | strmatch(rxdrgnam,"LITHON*") | strmatch(rxdrgnam,"LITHOT*") | strmatch(rxdrgnam,"ESKA*") | strmatch(rxdrgnam,"LAMICT*") | strmatch(rxdrgnam,"LAMOTR*") | strmatch(rxdrgnam,"OXTELL*") | strmatch(rxdrgnam,"TRILEPT*") | strmatch(rxdrgnam,"ZONIDAM*") | strmatch(rxdrgnam,"ZONEG*")

replace drug="antipsychotic" if strmatch(rxdrgnam,"ARIPIPRAZ*") | strmatch(rxdrgnam,"ABILIFY*") | strmatch(rxdrgnam,"ASENAP*") | strmatch(rxdrgnam,"SAPHRIS*") | strmatch(rxdrgnam,"BREXP*") | strmatch(rxdrgnam,"REXULT*") | strmatch(rxdrgnam,"CARIPRAZ*") | strmatch(rxdrgnam,"CHLORPROM*") | strmatch(rxdrgnam,"PROMAPAR*") | strmatch(rxdrgnam,"SONAZ*") | strmatch(rxdrgnam,"THORAZ*") | strmatch(rxdrgnam,"CLOZA*") | strmatch(rxdrgnam,"FAZACLO*") | strmatch(rxdrgnam,"VERSACLOZ*") | strmatch(rxdrgnam,"FLUPH*") | strmatch(rxdrgnam,"PERMITIL*") | strmatch(rxdrgnam,"PROLIXIN*") | strmatch(rxdrgnam,"FANAPT*") | strmatch(rxdrgnam,"ILOPERI*") | strmatch(rxdrgnam,"ZIPRAS*") | strmatch(rxdrgnam,"GEODON*") | strmatch(rxdrgnam,"HALOP*") | strmatch(rxdrgnam,"HALDO*") /| strmatch(rxdrgnam,"PALIP*") | strmatch(rxdrgnam,"INVEGA*") | strmatch(rxdrgnam,"LOXEP*") | strmatch(rxdrgnam,"LOXETA*") | strmatch(rxdrgnam,"ADASUV*") | strmatch(rxdrgnam,"LURAS*") | strmatch(rxdrgnam,"LATUDA*") | strmatch(rxdrgnam,"MOLIND*") | strmatch(rxdrgnam,"MOBAN") | strmatch(rxdrgnam,"THIOTH*") | strmatch(rxdrgnam,"NAVANE*") | strmatch(rxdrgnam,"OLANZ*") | strmatch(rxdrgnam,"SYMBYAX*") | strmatch(rxdrgnam,"ZYPREXA*") | strmatch(rxdrgnam,"PERPHEN*") | strmatch(rxdrgnam,"ETRAFON*") | strmatch(rxdrgnam,"TRIAVIL*") | strmatch(rxdrgnam,"TRILAFON*") | strmatch(rxdrgnam,"PIMAV*") | strmatch(rxdrgnam,"NUPLAZ*") | strmatch(rxdrgnam,"QUETIA*") | strmatch(rxdrgnam,"SEROQUE*") | strmatch(rxdrgnam,"RISPER*") | strmatch(rxdrgnam,"THIORI*") | strmatch(rxdrgnam,"MELLARIL*")

drop linkidx rxdrgnam

duplicates drop dupersid drug, force

gen byte n=1

reshape wide n, i(dupersid) j(drug) string

replace nantipsychotic=0 if nantipsychotic==.

replace nmoodstabilizer=0 if nmoodstabilizer==.

replace nbenzo=0 if nbenzo==.

save prescription2015.dta, replace

clear

use demographic_dignosis2015.dta

drop _merge

merge 1:m dupersid using prescription2015.dta

//variables definition

gen adult=0

replace adult=1 if age15x>=18

gen agecat=0

replace agecat=1 if age15x>=18 & age15x<30

replace agecat=2 if age15x>=30 & age15x<40

replace agecat=3 if age15x>=40 & age15x<50

replace agecat=4 if age15x>=50 & age15x<60

replace agecat=5 if age15x>=60

gen race=0

replace race=1 if racethx==2 //white

replace race=2 if racethx==3 //black

replace race=3 if racethx==1 //hispanic

gen marital=0

replace marital=1 if marry15x==1 //married

replace marital=2 if marry15x==2 | marry15x==3 | marry15x==4 //others

replace marital=3 if marry15x==5 //never married

gen education=0

replace education=1 if edrecode==1 | edrecode==2 //less than high school

replace education=2 if edrecode==13 | edrecode==14 //high school

replace education=3 if edrecode==15 | edrecode==16 //bachelor or higher

gen chronicdz=0

replace chronicdz=1 if hibpdx==1 | chddx==1 | strkdx==1 | diabdx==1

replace bmindx53=. if bmindx53==-1 | bmindx53==-9

gen bmicat=0

replace bmicat=1 if bmindx53<18.5 & bmindx53!=.

replace bmicat=2 if bmindx53>=18.5 & bmindx53<25

replace bmicat=3 if bmindx53>=25 & bmindx53<30

replace bmicat=4 if bmindx53>=30 & bmindx53!=.

replace k6sum42=. if k6sum42==-1 | k6sum42==-9

gen k6cat=0

replace k6cat=1 if k6sum42>=0 & k6sum42<13

replace k6cat=2 if k6sum42>=13 & k6sum42!=.

replace phq242=. if phq242==-1 | phq242==-9

gen phqcat=0

replace phqcat=1 if phq242>=0 & phq242<3

replace phqcat=2 if phq242>=3 & phq242!=.

gen famincomecat=0

replace famincomecat=1 if povcat15==1

replace famincomecat=2 if povcat15==2 | povcat15==3

replace famincomecat=3 if povcat15==4

replace famincomecat=4 if povcat15==5

//general information + prescription pattern

foreach x in namit nbupr ncita ndesv ndoxe ndulo nesci nfluo nmirt nnort nparo nsert nssnri ntca ntraz nvenl nvila {

gen `x'1=0

replace `x'1=1 if `x'==1

}

gen drugcount=namit1 + nbupr1 + ncita1 + ndesv1 + ndoxe1 + ndulo1 + nesci1 + nfluo1 + nmirt1 + nnort1 + nparo1 + nsert1 + ntraz1 + nvenl1 + nvila1 + nssnri1 + ntca1

gen drugcat=0

replace drugcat=1 if drugcount==1

replace drugcat=2 if drugcount>=2

gen antidepressanttype=0

replace antidepressanttype=1 if namit1==1 | ndoxe1==1 | nnort1==1 | ntca1==1

replace antidepressanttype=2 if ncita1==1 | nesci1==1 | nfluo1==1 | nparo1==1 | nsert1==1

replace antidepressanttype=3 if ndesv1==1 | ndulo1==1 | nvenl1==1 | nssnri1==1

replace antidepressanttype=4 if nbupr1==1 | nmirt1==1 | nvila1==1 | ntraz1==1

gen adultdepression=0

replace adultdepression=1 if depression==1 & adult==1

gen adultdepressionmono=0

replace adultdepressionmono=1 if depression==1 & adult==1 & drugcount==1

save demographic_dignosis_prescription2015.dta, replace

//general information

svyset [pweight=perwt15f], strata(varstr) psu(varpsu)

svy, subpop (if adult==1): tabulate adultdepression, format(%11.3g) count ci deff deft

svy, subpop (if adultdepression==1): tabulate drugcat

svy, subpop (if adultdepression==1): tabulate drugcat, format(%11.3g) count ci deff deft

svy, subpop (if adultdepressionmono==1): tabulate region15

svy, subpop (if adultdepressionmono==1): tabulate region15, format(%11.3g) count ci deff deft

svy, subpop (if adultdepressionmono==1): tabulate sex

svy, subpop (if adultdepressionmono==1): tabulate sex, format(%11.3g) count ci deff deft

summarize age15x [aweight=perwt15f] if adultdepressionmono==1, detail

svy, subpop (if adultdepressionmono==1): tabulate agecat

svy, subpop (if adultdepressionmono==1): tabulate agecat, format(%11.3g) count ci deff deft

svy, subpop (if adultdepressionmono==1): tabulate race

svy, subpop (if adultdepressionmono==1): tabulate race, format(%11.3g) count ci deff deft

svy, subpop (if adultdepressionmono==1): tabulate marital

svy, subpop (if adultdepressionmono==1): tabulate marital, format(%11.3g) count ci deff deft

svy, subpop (if adultdepressionmono==1): tabulate education

svy, subpop (if adultdepressionmono==1): tabulate education, format(%11.3g) count ci deff deft

svy, subpop (if adultdepressionmono==1): tabulate chronicdz

svy, subpop (if adultdepressionmono==1): tabulate chronicdz, format(%11.3g) count ci deff deft

//exlore the missing values of chronicdz

svy, subpop (if adultdepressionmono==1): tabulate hibpdx

svy, subpop (if adultdepressionmono==1): tabulate chddx

svy, subpop (if adultdepressionmono==1): tabulate strkdx

svy, subpop (if adultdepressionmono==1): tabulate cancerdx

svy, subpop (if adultdepressionmono==1): tabulate diabdx

svy, subpop (if adultdepressionmono==1): tabulate arthdx

gen chronicdzmissing=0

replace chronicdzmissing=1 if hibpdx==-8 | chddx==-8 | strkdx==-8 | diabdx==-8 | hibpdx==-9 | chddx==-9 | strkdx==-9 | diabdx==-9 | hibpdx==-7 | chddx==-7 | strkdx==-7 | diabdx==-7 | hibpdx==-1 | chddx==-1 | strkdx==-1 | diabdx==-1

svy, subpop(if adultdepressionmono==1): tabulate chronicdzmissing

summarize bmindx53 [aweight=perwt15f] if adultdepressionmono==1, detail

svy, subpop (if adultdepressionmono==1): tabulate bmicat

svy, subpop (if adultdepressionmono==1): tabulate bmicat, format(%11.3g) count ci deff deft

svy, subpop(if adultdepressionmono==1): tabulate adpain42

svy, subpop(if adultdepressionmono==1): tabulate adpain42, format(%11.3g) count ci deff deft

summarize k6sum42 [aweight=perwt15f] if adultdepressionmono==1, detail

svy, subpop (if adultdepressionmono==1): tabulate k6cat

svy, subpop (if adultdepressionmono==1): tabulate k6cat, format(%11.3g) count ci deff deft

summarize phq242 [aweight=perwt15f] if adultdepressionmono==1, detail

svy, subpop (if adultdepressionmono==1): tabulate phqcat

svy, subpop (if adultdepressionmono==1): tabulate phqcat, format(%11.3g) count ci deff deft

summarize faminc15 [aweight=perwt15f] if adultdepressionmono==1, detail

svy, subpop (if adultdepressionmono==1): tabulate famincomecat

svy, subpop (if adultdepressionmono==1): tabulate famincomecat, format(%11.3g) count ci deff deft

svy, subpop (if adultdepressionmono==1): tabulate inscov15

svy, subpop (if adultdepressionmono==1): tabulate inscov15, format(%11.3g) count ci deff deft

svy, subpop (if adultdepressionmono==1): tabulate nbenzo

svy, subpop (if adultdepressionmono==1): tabulate nbenzo, format(%11.3g) count ci deff deft

svy, subpop (if adultdepressionmono==1): tabulate nantipsychotic

svy, subpop (if adultdepressionmono==1): tabulate nantipsychotic, format(%11.3g) count ci deff deft

svy, subpop (if adultdepressionmono==1): tabulate nmoodstabilizer

svy, subpop (if adultdepressionmono==1): tabulate nmoodstabilizer, format(%11.3g) count ci deff deft

//prescription pattern

svy, subpop (if adultdepressionmono==1): tabulate antidepressanttype

svy, subpop (if adultdepressionmono==1): tabulate antidepressanttype, format(%11.3g) count ci deff deft

svy, subpop (if adultdepressionmono==1): proportion namit1

svy, subpop (if adultdepressionmono==1): tabulate namit1, format(%11.3g) count ci deff deft

svy, subpop (if adultdepressionmono==1): proportion nbupr1

svy, subpop (if adultdepressionmono==1): tabulate nbupr1, format(%11.3g) count ci deff deft

svy, subpop (if adultdepressionmono==1): proportion ncita1

svy, subpop (if adultdepressionmono==1): tabulate ncita1, format(%11.3g) count ci deff deft

svy, subpop (if adultdepressionmono==1): proportion ndesv1

svy, subpop (if adultdepressionmono==1): tabulate ndesv1, format(%11.3g) count ci deff deft

svy, subpop (if adultdepressionmono==1): proportion ndoxe1

svy, subpop (if adultdepressionmono==1): tabulate ndoxe1, format(%11.3g) count ci deff deft

svy, subpop (if adultdepressionmono==1): proportion ndulo1

svy, subpop (if adultdepressionmono==1): tabulate ndulo1, format(%11.3g) count ci deff deft

svy, subpop (if adultdepressionmono==1): proportion nesci1

svy, subpop (if adultdepressionmono==1): tabulate nesci1, format(%11.3g) count ci deff deft

svy, subpop (if adultdepressionmono==1): proportion nfluo1

svy, subpop (if adultdepressionmono==1): tabulate nfluo1, format(%11.3g) count ci deff deft

svy, subpop (if adultdepressionmono==1): proportion nmirt1

svy, subpop (if adultdepressionmono==1): tabulate nmirt1, format(%11.3g) count ci deff deft

svy, subpop (if adultdepressionmono==1): proportion nnort1

svy, subpop (if adultdepressionmono==1): tabulate nnort1, format(%11.3g) count ci deff deft

svy, subpop (if adultdepressionmono==1): proportion nparo1

svy, subpop (if adultdepressionmono==1): tabulate nparo1, format(%11.3g) count ci deff deft

svy, subpop (if adultdepressionmono==1): proportion nsert1

svy, subpop (if adultdepressionmono==1): tabulate nsert1, format(%11.3g) count ci deff deft

svy, subpop (if adultdepressionmono==1): proportion ntraz1

svy, subpop (if adultdepressionmono==1): tabulate ntraz1, format(%11.3g) count ci deff deft

svy, subpop (if adultdepressionmono==1): proportion nvenl1

svy, subpop (if adultdepressionmono==1): tabulate nvenl1, format(%11.3g) count ci deff deft

svy, subpop (if adultdepressionmono==1): proportion nvila1

svy, subpop (if adultdepressionmono==1): tabulate nvila1, format(%11.3g) count ci deff deft

//average daily doses

clear

use "prescription dta in 2015"

keep dupersid linkidx purchrd rxbegyrx rxdrgnam rxstreng rxquanty rxdaysup

duplicates drop linkidx, force

//unite the drug name & focus on ADs/benzo/mood stabilizers/antipsychotics

gen drug="others"

replace drug="amit" if strmatch(rxdrgnam,"AMITR*") | strmatch(rxdrgnam,"AMITID*") | strmatch(rxdrgnam,"ELAVIL*") | strmatch(rxdrgnam,"ENDEP")

replace drug="amox" if strmatch(rxdrgnam,"AMOXA*") | strmatch(rxdrgnam,"ASENDIN*")

replace drug="bupr" if strmatch(rxdrgnam,"BUPROP*") | strmatch(rxdrgnam,"APLENZIN*") | strmatch(rxdrgnam,"FORFIVO*") | strmatch(rxdrgnam,"WELLBUTRIN*") | strmatch(rxdrgnam,"ZYBAN") | strmatch(rxdrgnam,"BUPROBAN") | strmatch(rxdrgnam,"BUDEPRON")

replace drug="cita" if strmatch(rxdrgnam,"CITALO*") | strmatch(rxdrgnam,"CELEXA*")

replace drug="clom" if strmatch(rxdrgnam,"CLOMIPRAM*") | strmatch(rxdrgnam,"ANAFRANIL*")

replace drug="desi" if strmatch(rxdrgnam,"DESIPRAM*") | strmatch(rxdrgnam,"NORPRAMIN*") | strmatch(rxdrgnam,"PERTOFRANE*")

replace drug="desv" if strmatch(rxdrgnam,"DESVENLA*") | strmatch(rxdrgnam,"KHEDEZLA*") | strmatch(rxdrgnam,"PRISTQ*")

replace drug="doxe" if strmatch(rxdrgnam,"DOXEPIN*") | strmatch(rxdrgnam,"SILENOR*") | strmatch(rxdrgnam,"SINEQUAN*") | strmatch(rxdrgnam,"ZONALON*")

replace drug="dulo" if strmatch(rxdrgnam,"DULOXE*") | strmatch(rxdrgnam,"CYMBALTA*")

replace drug="esci" if strmatch(rxdrgnam,"ESCITALOP*") | strmatch(rxdrgnam,"LEXAPRO*")

replace drug="fluo" if strmatch(rxdrgnam,"FLUOXE*") | strmatch(rxdrgnam,"FLUOEXT*") | strmatch(rxdrgnam,"PROZAC*") | strmatch(rxdrgnam,"PROSAC") | strmatch(rxdrgnam,"SARAFEM*") | strmatch(rxdrgnam,"SELFEMRA*")

replace drug="imip" if strmatch(rxdrgnam,"IMIPRAM*") | strmatch(rxdrgnam,"PRAMINE*") | strmatch(rxdrgnam,"PRESAMINE*") | strmatch(rxdrgnam,"TOFRANIL*")

replace drug="levo" if strmatch(rxdrgnam,"LEVOMILNACIP*") | strmatch(rxdrgnam,"FETZIMA*")

replace drug="mirt" if strmatch(rxdrgnam,"MIRTAZ*") | strmatch(rxdrgnam,"REMERON*")

replace drug="nefa" if strmatch(rxdrgnam,"NEFAZO*") | strmatch(rxdrgnam,"SERZONE*")

replace drug="nort" if strmatch(rxdrgnam,"NORTRIP*") | strmatch(rxdrgnam,"AVENTYL*") | strmatch(rxdrgnam,"PAMELOR*")

replace drug="paro" if strmatch(rxdrgnam,"PAROXE*") | strmatch(rxdrgnam,"BRISDELLE*") | strmatch(rxdrgnam,"PAXIL*") | strmatch(rxdrgnam,"PEXEVA*")

replace drug="prot" if strmatch(rxdrgnam,"PROTRIP*") | strmatch(rxdrgnam,"VIVACTIL*")

replace drug="sert" if strmatch(rxdrgnam,"SERTR*") | strmatch(rxdrgnam,"ZOLOFT*")

replace drug="traz" if strmatch(rxdrgnam,"TRAZO*") | strmatch(rxdrgnam,"TRAZAD*") | strmatch(rxdrgnam,"DESYREL*") | strmatch(rxdrgnam,"TRIALODIN*")

replace drug="trim" if strmatch(rxdrgnam,"TRIMIPRAM*") | strmatch(rxdrgnam,"SURMONTIL*")

replace drug="venl" if strmatch(rxdrgnam,"VENLAF*") | strmatch(rxdrgnam,"EFFEXOR*")

replace drug="vila" if strmatch(rxdrgnam,"VILAZOD*") | strmatch(rxdrgnam,"VIIBRYD*")

replace drug="vort" if strmatch(rxdrgnam,"VORTIOX*")

replace drug="benzo" if strmatch(rxdrgnam,"BENZODIAZ*") | strmatch(rxdrgnam,"ALPRAZ*") | strmatch(rxdrgnam,"NIRAVAM*") | strmatch(rxdrgnam,"XANAX*") | strmatch(rxdrgnam,"CHLORDIA*") | strmatch(rxdrgnam,"A-POXIDE*") | strmatch(rxdrgnam,"LIBR*") | strmatch(rxdrgnam,"LIMBI*") | strmatch(rxdrgnam,"LYGEN") | strmatch(rxdrgnam,"MENRIUM*") | strmatch(rxdrgnam,"CLOBA*") | strmatch(rxdrgnam,"ONFI") | strmatch(rxdrgnam,"SIMPAZ*") | strmatch(rxdrgnam,"CLONAZEPAM") | strmatch(rxdrgnam,"KLONOPIN*") | strmatch(rxdrgnam,"CLORAZEP*") | strmatch(rxdrgnam,"GEN-XENE") | strmatch(rxdrgnam,"TRANXENE") | strmatch(rxdrgnam,"DIAZEPAM*") | strmatch(rxdrgnam,"DIASTAT*") | strmatch(rxdrgnam,"DIZAC") | strmatch(rxdrgnam,"Q-PAM") | strmatch(rxdrgnam,"VALIUM*") | strmatch(rxdrgnam,"VALRELEASE*") | strmatch(rxdrgnam,"ESTAZOLAM*") | strmatch(rxdrgnam,"PROSOM") strmatch(rxdrgnam,"FLURAZEPAM*") | strmatch(rxdrgnam,"DALMANE*") | strmatch(rxdrgnam,"HALAZEPAM*") | strmatch(rxdrgnam,"PAXIPAM*") | strmatch(rxdrgnam,"LORAZ*") | strmatch(rxdrgnam,"ATIVAN") | strmatch(rxdrgnam,"MIDAZOLAM*") | strmatch(rxdrgnam,"SEIZALAM*") | strmatch(rxdrgnam,"VERSED") | strmatch(rxdrgnam,"OXAZEPAM*") | strmatch(rxdrgnam,"SERAX")| strmatch(rxdrgnam,"ZAXOPAM*") | strmatch(rxdrgnam,"QUAZEPAM*") | strmatch(rxdrgnam,"DORAL*") | strmatch(rxdrgnam,"TEMAZ*") | strmatch(rxdrgnam,"RESTORIL*") | strmatch(rxdrgnam,"TRIAZOLAM*") | strmatch(rxdrgnam,"HALCION*") | strmatch(rxdrgnam,"FLUMAZEN*") | strmatch(rxdrgnam,"ROMAZI") | strmatch(rxdrgnam,"ESZOPI*") | strmatch(rxdrgnam,"LUNESTA*") | strmatch(rxdrgnam,"ZALEPLON*") | strmatch(rxdrgnam,"SONATA*") | strmatch(rxdrgnam,"ZOLPI*") | strmatch(rxdrgnam,"AMBIEN") | strmatch(rxdrgnam,"EDLUAR*") | strmatch(rxdrgnam,"INTERMEZ*") | strmatch(rxdrgnam,"TOVALT*")

replace drug="moodstabilizer" if strmatch(rxdrgnam,"*CARBAMAZ*") | strmatch(rxdrgnam,"CARBATROL*") | strmatch(rxdrgnam,"CARNEXIV*") | strmatch(rxdrgnam,"EPITOL*") | strmatch(rxdrgnam,"EQUETRO*") | strmatch(rxdrgnam,"TERIL") | strmatch(rxdrgnam,"TEGRETOL*") | strmatch(rxdrgnam,"VALPRO*") | strmatch(rxdrgnam,"DEPACON*") | strmatch(rxdrgnam,"DEPAK*") | strmatch(rxdrgnam,"STAVZOR*") | strmatch(rxdrgnam,"DIVALP*") | strmatch(rxdrgnam,"LITHIU*") | strmatch(rxdrgnam,"LITHANE*") | strmatch(rxdrgnam,"LITHOB*") | strmatch(rxdrgnam,"LITHON*") | strmatch(rxdrgnam,"LITHOT*") | strmatch(rxdrgnam,"ESKA*") | strmatch(rxdrgnam,"LAMICT*") | strmatch(rxdrgnam,"LAMOTR*") | strmatch(rxdrgnam,"OXTELL*") | strmatch(rxdrgnam,"TRILEPT*") | strmatch(rxdrgnam,"ZONIDAM*") | strmatch(rxdrgnam,"ZONEG*")

replace drug="antipsychotic" if strmatch(rxdrgnam,"ARIPIPRAZ*") | strmatch(rxdrgnam,"ABILIFY*") | strmatch(rxdrgnam,"ASENAP*") | strmatch(rxdrgnam,"SAPHRIS*") | strmatch(rxdrgnam,"BREXP*") | strmatch(rxdrgnam,"REXULT*") | strmatch(rxdrgnam,"CARIPRAZ*") | strmatch(rxdrgnam,"CHLORPROM*") | strmatch(rxdrgnam,"PROMAPAR*") | strmatch(rxdrgnam,"SONAZ*") | strmatch(rxdrgnam,"THORAZ*") | strmatch(rxdrgnam,"CLOZA*") | strmatch(rxdrgnam,"FAZACLO*") | strmatch(rxdrgnam,"VERSACLOZ*") | strmatch(rxdrgnam,"FLUPH*") | strmatch(rxdrgnam,"PERMITIL*") | strmatch(rxdrgnam,"PROLIXIN*") | strmatch(rxdrgnam,"FANAPT*") | strmatch(rxdrgnam,"ILOPERI*") | strmatch(rxdrgnam,"ZIPRAS*") | strmatch(rxdrgnam,"GEODON*") | strmatch(rxdrgnam,"HALOP*") | strmatch(rxdrgnam,"HALDO*") /| strmatch(rxdrgnam,"PALIP*") | strmatch(rxdrgnam,"INVEGA*") | strmatch(rxdrgnam,"LOXEP*") | strmatch(rxdrgnam,"LOXETA*") | strmatch(rxdrgnam,"ADASUV*") | strmatch(rxdrgnam,"LURAS*") | strmatch(rxdrgnam,"LATUDA*") | strmatch(rxdrgnam,"MOLIND*") | strmatch(rxdrgnam,"MOBAN") | strmatch(rxdrgnam,"THIOTH*") | strmatch(rxdrgnam,"NAVANE*") | strmatch(rxdrgnam,"OLANZ*") | strmatch(rxdrgnam,"SYMBYAX*") | strmatch(rxdrgnam,"ZYPREXA*") | strmatch(rxdrgnam,"PERPHEN*") | strmatch(rxdrgnam,"ETRAFON*") | strmatch(rxdrgnam,"TRIAVIL*") | strmatch(rxdrgnam,"TRILAFON*") | strmatch(rxdrgnam,"PIMAV*") | strmatch(rxdrgnam,"NUPLAZ*") | strmatch(rxdrgnam,"QUETIA*") | strmatch(rxdrgnam,"SEROQUE*") | strmatch(rxdrgnam,"RISPER*") | strmatch(rxdrgnam,"THIORI*") | strmatch(rxdrgnam,"MELLARIL*")

duplicates drop dupersid drug, force

save prescription2015fordosecomplete.dta, replace

drop rxbegyrx rxdrgnam rxstreng purchrd

gen byte n=1

reshape wide n, i(linkidx) j(drug) string

save prescription2015fordosepart.dta, replace

clear

use “prescription2015fordosecomplete.dta”

merge 1:1 linkidx using prescription2015fordosepart.dta

drop purchrd _merge

save prescription2015fordose.dta, replace

clear

use "demographic_dignosis2015.dta"

drop _merge

merge 1:m dupersid using prescription2015fordose.dta

sort dupersid

foreach x in namit nbupr ncita ndesv ndoxe ndulo nesci nmirt nnort nparo nsert ntraz nvenl nvila {

gen `x'1=0

replace `x'1=1 if `x'==1

}

gen drugcount=namit1 + nbupr1 + ncita1 + ndesv1 + ndoxe1 + ndulo1 + nesci1 + nfluo1 + nmirt1 + nnort1 + nparo1 + nsert1 + ntraz1 + nvenl1 + nvila1

egen truedrugcount=sum(drugcount), by(dupersid)

gen truedrugcat=0

replace truedrugcat=1 if truedrugcount==1

replace truedrugcat=2 if truedrugcount>=2

replace nantipsychotic=0 if nantipsychotic==.

replace nmoodstabilizer=0 if nmoodstabilizer==.

replace nbenzo=0 if nbenzo==.

//variables definition

gen adult=0

replace adult=1 if age15x>=18

gen agecat=0

replace agecat=1 if age15x>=18 & age15x<30

replace agecat=2 if age15x>=30 & age15x<40

replace agecat=3 if age15x>=40 & age15x<50

replace agecat=4 if age15x>=50 & age15x<60

replace agecat=5 if age15x>=60

gen age10=age15x/10

gen race=0

replace race=1 if racethx==2

replace race=2 if racethx==3

replace race=3 if racethx==1

gen region=.

replace region=region15 if region15>=1

gen marital=0

replace marital=1 if marry15x==1

replace marital=2 if marry15x==2 | marry15x==3 | marry15x==4

replace marital=3 if marry15x==5

gen education=.

replace education=1 if edrecode==1 | edrecode==2

replace education=2 if edrecode==13 | edrecode==14

replace education=3 if edrecode==15 | edrecode==16

gen chronicdz=0

replace chronicdz=1 if hibpdx==1 | chddx==1 | strkdx==1 | diabdx==1

gen cancer=.

replace cancer=1 if cancerdx==1

replace cancer=0 if cancerdx==2

replace bmindx53=. if bmindx53==-1 | bmindx53==-9

gen bmicat=.

replace bmicat=1 if bmindx53<18.5

replace bmicat=2 if bmindx53>=18.5 & bmindx53<25

replace bmicat=3 if bmindx53>=25 & bmindx53<30

replace bmicat=4 if bmindx53>=30 & bmindx53!=.

gen adpain=.

replace adpain=adpain42 if adpain42>=1

gen pain=.

replace pain=0 if adpain42>=1 & adpain42<4

replace pain=1 if adpain42>=4

replace k6sum42=. if k6sum42==-1 | k6sum42==-9

gen k6cat=.

replace k6cat=1 if k6sum42>=0 & k6sum42<13

replace k6cat=2 if k6sum42>=13 & k6sum42!=.

replace phq242=. if phq242==-1 | phq242==-9

gen phqcat=.

replace phqcat=1 if phq242>=0 & phq242<3

replace phqcat=2 if phq242>=3 & phq242!=.

gen faminc10000=faminc15/10000

gen famincomecat=0

replace famincomecat=1 if povcat15==1

replace famincomecat=2 if povcat15==2 | povcat15==3

replace famincomecat=3 if povcat15==4

replace famincomecat=4 if povcat15==5

gen antidepressanttype=0

replace antidepressanttype=1 if namit1==1 | ndoxe1==1 | nnort1==1

replace antidepressanttype=2 if ncita1==1 | nesci1==1 | nfluo1==1 | nparo1==1 | nsert1==1

replace antidepressanttype=3 if ndesv1==1 | ndulo1==1 | nvenl1==1

replace antidepressanttype=4 if nbupr1==1 | nmirt1==1 | nvila1==1 | ntraz1==1

gen adultdepression=0

replace adultdepression=1 if depression==1 & adult==1

gen antidepressant=0

replace antidepressant=1 if namit1==1 | nbupr1==1 | ncita1==1 | ndesv1==1 | ndoxe1==1 | ndulo1==1 | nesci1==1 | nfluo1==1 | nmirt1==1 | nnort1==1 | nparo1==1 | nsert1==1 | ntraz1==1 | nvenl1==1 | nvila1==1

gen adultdepressionmono=0

replace adultdepressionmono=1 if depression==1 & adult==1 & truedrugcount==1 & antidepressant==1

//dose (low dosage) and duration definition

destring rxstreng, replace force

replace rxstreng=. if rxstreng==-9

destring rxdaysup, replace force

replace rxdaysup=. if rxdaysup==-8 | rxdaysup==-7 | rxdaysup==0

gen dosage=.

replace dosage=rxquanty*rxstreng/rxdaysup

replace rxbegyrx=. if rxbegyrx==-1 | rxbegyrx==-7 | rxbegyrx==-8 | rxbegyrx==-9 | rxbegyrx==-14

gen duration=.

replace duration=2015-rxbegyrx

gen durationcat=.

replace durationcat=1 if duration<=1 & duration!=.

replace durationcat=2 if duration>=2 & duration<=4

replace durationcat=3 if duration>=5 & duration!=.

gen lowdosage=.

replace lowdosage=1 if dosage>0 & dosage<50 & namit1==1 //amit 50-300mg

replace lowdosage=0 if dosage>=50 & dosage!=. & namit1==1

replace lowdosage=1 if dosage>0 & dosage<300 & nbupr1==1 //bupr 300-450mg

replace lowdosage=0 if dosage>=300 & dosage!=. & nbupr1==1

replace lowdosage=1 if dosage>0 & dosage<20 & ncita1==1 //cita 20-40mg

replace lowdosage=0 if dosage>=20 & dosage!=. & ncita1==1

replace lowdosage=1 if dosage>0 & dosage<50 & ndesv1==1 //desv 50-100mg

replace lowdosage=0 if dosage>=50 & dosage!=. & ndesv1==1

replace lowdosage=1 if dosage>0 & dosage<75 & ndoxe1==1 //doxe 75-300mg

replace lowdosage=0 if dosage>=75 & dosage!=. & ndoxe1==1

replace lowdosage=1 if dosage>0 & dosage<40 & ndulo1==1 //dulo 40-120mg

replace lowdosage=0 if dosage>=40 & dosage!=. & ndulo1==1

replace lowdosage=1 if dosage>0 & dosage<10 & nesci1==1 //esci 10-20mg

replace lowdosage=0 if dosage>=10 & dosage!=. & nesci1==1

replace lowdosage=1 if dosage>0 & dosage<20 & nfluo1==1 //fluo 20-80mg

replace lowdosage=0 if dosage>=20 & dosage!=. & nfluo1==1

replace lowdosage=1 if dosage>0 & dosage<15 & nmirt1==1 //mirt 15-45mg

replace lowdosage=0 if dosage>=15 & dosage!=. & nmirt1==1

replace lowdosage=1 if dosage>0 & dosage<75 & nnort1==1 //nort 75-150mg

replace lowdosage=0 if dosage>=75 & dosage!=. & nnort1==1

replace lowdosage=1 if dosage>0 & dosage<20 & nparo1==1 //paro 20-50mg

replace lowdosage=0 if dosage>=20 & dosage!=. & nparo1==1

replace lowdosage=1 if dosage>0 & dosage<50 & nsert1==1 //sert 50-200mg

replace lowdosage=0 if dosage>=50 & dosage!=. & nsert1==1

replace lowdosage=1 if dosage>0 & dosage<150 & ntraz1==1 //traz 150-400mg

replace lowdosage=0 if dosage>=150 & dosage!=. & ntraz1==1

replace lowdosage=1 if dosage>0 & dosage<75 & nvenl1==1 //venl 75-375mg

replace lowdosage=0 if dosage>=75 & dosage!=. & nvenl1==1

replace lowdosage=1 if dosage>0 & dosage<20 & nvila1==1 //vila 20-40mg

replace lowdosage=0 if dosage>=20 & dosage!=. & nvila1==1

//average dosage

svyset [pweight=perwt15f], strata(varstr) psu(varpsu)

summarize dosage [aweight=perwt15f] if adultdepressionmono==1 & namit1==1, detail

svy, subpop (if adultdepressionmono==1 & namit1==1): mean dosage

estat sd

summarize dosage [aweight=perwt15f] if adultdepressionmono==1 & nbupr1==1, detail

svy, subpop(if adultdepressionmono==1 & nbupr1==1): mean dosage

estat sd

summarize dosage [aweight=perwt15f] if adultdepressionmono==1 & ncita1==1, detail

svy, subpop (if adultdepressionmono==1 & ncita1==1): mean dosage

estat sd

summarize dosage [aweight=perwt15f] if adultdepressionmono==1 & ndesv1==1, detail

svy, subpop (if adultdepressionmono==1 & ndesv1==1): mean dosage

estat sd

summarize dosage [aweight=perwt15f] if adultdepressionmono==1 & ndulo1==1, detail

svy, subpop (if adultdepressionmono==1 & ndulo1==1): mean dosage

estat sd

summarize dosage [aweight=perwt15f] if adultdepressionmono==1 & nesci1==1, detail

svy, subpop (if adultdepressionmono==1 & nesci1==1): mean dosage

estat sd

summarize dosage [aweight=perwt15f] if adultdepressionmono==1 & nfluo1==1, detail

svy, subpop (if adultdepressionmono==1 & nfluo1==1): mean dosage

estat sd

summarize dosage [aweight=perwt15f] if adultdepressionmono==1 & nmirt1==1, detail

svy, subpop (if adultdepressionmono==1 & nmirt1==1): mean dosage

estat sd

summarize dosage [aweight=perwt15f] if adultdepressionmono==1 & nparo1==1, detail

svy, subpop (if adultdepressionmono==1 & nparo1==1): mean dosage

estat sd

summarize dosage [aweight=perwt15f] if adultdepressionmono==1 & nsert1==1, detail

svy, subpop (if adultdepressionmono==1 & nsert1==1): mean dosage

estat sd

summarize dosage [aweight=perwt15f] if adultdepressionmono==1 & ntraz1==1, detail

svy, subpop (if adultdepressionmono==1 & ntraz1==1): mean dosage

estat sd

summarize dosage [aweight=perwt15f] if adultdepressionmono==1 & nvenl1==1, detail

svy, subpop (if adultdepressionmono==1 & nvenl1==1): mean dosage

estat sd

summarize dosage [aweight=perwt15f] if adultdepressionmono==1 & nvila1==1, detail

svy, subpop (if adultdepressionmono==1 & nvila1==1): mean dosage

estat sd

//duration

summarize duration [aweight=perwt15f] if adultdepressionmono==1, detail

svy, subpop (if adultdepressionmono==1): tabulate durationcat, missing

svy, subpop (if adultdepressionmono==1): tabulate durationcat, format(%11.3g) count ci deff deft

//proportion of dosage lower than licensed range

svy, subpop(if adultdepressionmono==1): tabulate lowdosage, missing

svy, subpop(if adultdepressionmono==1): tabulate lowdosage, format(%11.3g) count ci deff deft

//characteristics of patients using low dosage

svy, subpop (if adultdepressionmono==1 & lowdosage==1): tabulate sex

summarize age15x [aweight=perwt15f] if adultdepressionmono==1 & lowdosage==1, detail

svy, subpop (if adultdepressionmono==1 & lowdosage==1): tabulate agecat

svy, subpop (if adultdepressionmono==1 & lowdosage==1): tabulate race

svy, subpop (if adultdepressionmono==1 & lowdosage==1): tabulate marital

svy, subpop (if adultdepressionmono==1 & lowdosage==1): tabulate education, missing

svy, subpop (if adultdepressionmono==1 & lowdosage==1): tabulate chronicdz

svy, subpop (if adultdepressionmono==1 & lowdosage==1): tabulate cancerdx

summarize bmindx53 [aweight=perwt15f] if adultdepressionmono==1 & lowdosage==1, detail

svy, subpop (if adultdepressionmono==1 & lowdosage==1): tabulate bmicat, missing

svy, subpop (if adultdepressionmono==1 & lowdosage==1): tabulate adpain42, missing

summarize pcs42 [aweight=perwt15f] if adultdepressionmono==1 & lowdosage==1, detail

summarize mcs42 [aweight=perwt15f] if adultdepressionmono==1 & lowdosage==1, detail

summarize k6sum42 [aweight=perwt15f] if adultdepressionmono==1 & lowdosage==1, detail

svy, subpop (if adultdepressionmono==1 & lowdosage==1): tabulate k6cat, missing

summarize phq242 [aweight=perwt15f] if adultdepressionmono==1 & lowdosage==1, detail

svy, subpop (if adultdepressionmono==1 & lowdosage==1): tabulate phqcat, missing

summarize faminc15 [aweight=perwt15f] if adultdepressionmono==1 & lowdosage==1, detail

svy, subpop (if adultdepressionmono==1 & lowdosage==1): tabulate famincomecat

svy, subpop (if adultdepressionmono==1 & lowdosage==1): tabulate inscov15

svy, subpop (if adultdepressionmono==1 & lowdosage==1): tabulate nbenzo

svy, subpop (if adultdepressionmono==1 & lowdosage==1): tabulate nantipsychotic

svy, subpop (if adultdepressionmono==1 & lowdosage==1): tabulate nmoodstabilizer

svy, subpop (if adultdepressionmono==1 & lowdosage==1): tabulate antidepressanttype

summarize duration [aweight=perwt15f] if adultdepressionmono==1 & lowdosage==1, detail

svy, subpop (if adultdepressionmono==1 & lowdosage==1): tabulate durationcat, missing

//characteristics of patients using usual dosage

svy, subpop (if adultdepressionmono==1 & lowdosage==0): tabulate sex

summarize age15x [aweight=perwt15f] if adultdepressionmono==1 & lowdosage==0, detail

svy, subpop (if adultdepressionmono==1 & lowdosage==0): tabulate agecat

svy, subpop (if adultdepressionmono==1 & lowdosage==0): tabulate race

svy, subpop (if adultdepressionmono==1 & lowdosage==0): tabulate marital

svy, subpop (if adultdepressionmono==1 & lowdosage==0): tabulate education, missing

svy, subpop (if adultdepressionmono==1 & lowdosage==0): tabulate chronicdz

svy, subpop (if adultdepressionmono==1 & lowdosage==0): tabulate cancerdx

summarize bmindx53 [aweight=perwt15f] if adultdepressionmono==1 & lowdosage==0, detail

svy, subpop (if adultdepressionmono==1 & lowdosage==0): tabulate bmicat

svy, subpop (if adultdepressionmono==1 & lowdosage==0): tabulate adpain42, missing

summarize pcs42 [aweight=perwt15f] if adultdepressionmono==1 & lowdosage==0, detail

summarize mcs42 [aweight=perwt15f] if adultdepressionmono==1 & lowdosage==0, detail

summarize k6sum42 [aweight=perwt15f] if adultdepressionmono==1 & lowdosage==0, detail

svy, subpop (if adultdepressionmono==1 & lowdosage==0): tabulate k6cat, missing

summarize phq242 [aweight=perwt15f] if adultdepressionmono==1 & lowdosage==0, detail

svy, subpop (if adultdepressionmono==1 & lowdosage==0): tabulate phqcat, missing

summarize faminc15 [aweight=perwt15f] if adultdepressionmono==1 & lowdosage==0, detail

svy, subpop (if adultdepressionmono==1 & lowdosage==0): tabulate famincomecat

svy, subpop (if adultdepressionmono==1 & lowdosage==0): tabulate inscov15

svy, subpop (if adultdepressionmono==1 & lowdosage==0): tabulate nbenzo

svy, subpop (if adultdepressionmono==1 & lowdosage==0): tabulate nantipsychotic

svy, subpop (if adultdepressionmono==1 & lowdosage==0): tabulate nmoodstabilizer

svy, subpop (if adultdepressionmono==1 & lowdosage==0): tabulate antidepressanttype

summarize duration [aweight=perwt15f] if adultdepressionmono==1 & lowdosage==0, detail

svy, subpop (if adultdepressionmono==1 & lowdosage==0): tabulate durationcat, missing

//univariable regression

summarize age15x [aweight=perwt15f] if adultdepressionmono==1 & lowdosage!=., detail

svy, subpop (if adultdepressionmono==1 & lowdosage!=.): logistic lowdosage age10

svy, subpop (if adultdepressionmono==1 & lowdosage!=.): logistic lowdosage ib(1).agecat

svy, subpop (if adultdepressionmono==1 & lowdosage!=.): logistic lowdosage ib(1).sex

svy, subpop (if adultdepressionmono==1 & lowdosage!=.): tabulate region15

svy, subpop (if adultdepressionmono==1 & lowdosage!=.): logistic lowdosage ib(1).region

svy, subpop (if adultdepressionmono==1 & lowdosage!=.): logistic lowdosage ib(1).race

svy, subpop (if adultdepressionmono==1 & lowdosage!=.): logistic lowdosage ib(1).marital

svy, subpop (if adultdepressionmono==1 & lowdosage!=.): logistic lowdosage ib(1).education

svy, subpop (if adultdepressionmono==1 & lowdosage!=.): logistic lowdosage chronicdz

svy, subpop (if adultdepressionmono==1 & lowdosage!=.): tabulate cancerdx

svy, subpop (if adultdepressionmono==1 & lowdosage!=.): logistic lowdosage cancer

svy, subpop (if adultdepressionmono==1 & lowdosage!=.): tabulate bmicat

svy, subpop (if adultdepressionmono==1 & lowdosage!=.): logistic lowdosage ib(1).bmicat

svy, subpop (if adultdepressionmono==1 & lowdosage!=.): logistic lowdosage bmindx53

svy, subpop (if adultdepressionmono==1 & lowdosage!=.): logistic lowdosage faminc15

svy, subpop (if adultdepressionmono==1 & lowdosage!=.): logistic lowdosage faminc10000

svy, subpop (if adultdepressionmono==1 & lowdosage!=.): logistic lowdosage ib(1).famincomecat

svy, subpop (if adultdepressionmono==1 & lowdosage!=.): logistic lowdosage ib(1).inscov15

svy, subpop (if adultdepressionmono==1 & lowdosage!=.): tabulate durationcat

svy, subpop (if adultdepressionmono==1 & lowdosage!=.): logistic lowdosage ib(1).durationcat

svy, subpop (if adultdepressionmono==1 & lowdosage!=.): logistic lowdosage duration

svy, subpop (if adultdepressionmono==1 & lowdosage!=.): tabulate adpain42

svy, subpop (if adultdepressionmono==1 & lowdosage!=.): logistic lowdosage ib(1).adpain

svy, subpop (if adultdepressionmono==1 & lowdosage!=.): logistic lowdosage pain

svy, subpop (if adultdepressionmono==1 & lowdosage!=.): tabulate k6cat

svy, subpop (if adultdepressionmono==1 & lowdosage!=.): logistic lowdosage ib(1).k6cat

svy, subpop (if adultdepressionmono==1 & lowdosage!=.): logistic lowdosage k6sum42

svy, subpop (if adultdepressionmono==1 & lowdosage!=.): tabulate phqcat

svy, subpop (if adultdepressionmono==1 & lowdosage!=.): logistic lowdosage ib(1).phqcat

svy, subpop (if adultdepressionmono==1 & lowdosage!=.): logistic lowdosage phq242

svy, subpop (if adultdepressionmono==1 & lowdosage!=.): logistic lowdosage ib(1).antidepressanttype

//adjusted model for age sex bmi(cont)

svy, subpop (if adultdepressionmono==1 & lowdosage!=.): logistic lowdosage ib(1).agecat ib(1).sex bmindx53

svy, subpop (if adultdepressionmono==1 & lowdosage!=.): logistic lowdosage ib(1).race ib(1).agecat ib(1).sex bmindx53

svy, subpop (if adultdepressionmono==1 & lowdosage!=.): logistic lowdosage ib(1).education ib(1).agecat ib(1).sex bmindx53

svy, subpop (if adultdepressionmono==1 & lowdosage!=.): logistic lowdosage ib(1).marital ib(1).agecat ib(1).sex bmindx53

svy, subpop (if adultdepressionmono==1 & lowdosage!=.): logistic lowdosage faminc10000 ib(1).agecat ib(1).sex bmindx53

svy, subpop (if adultdepressionmono==1 & lowdosage!=.): logistic lowdosage ib(1).famincomecat ib(1).agecat ib(1).sex bmindx53

svy, subpop (if adultdepressionmono==1 & lowdosage!=.): logistic lowdosage ib(1).inscov15 ib(1).agecat ib(1).sex bmindx53

svy, subpop (if adultdepressionmono==1 & lowdosage!=.): logistic lowdosage phq242 ib(1).agecat ib(1).sex bmindx53

svy, subpop (if adultdepressionmono==1 & lowdosage!=.): logistic lowdosage ib(1).phqcat ib(1).agecat ib(1).sex bmindx53

svy, subpop (if adultdepressionmono==1 & lowdosage!=.): logistic lowdosage k6sum42 ib(1).agecat ib(1).sex bmindx53

svy, subpop (if adultdepressionmono==1 & lowdosage!=.): logistic lowdosage ib(1).k6cat ib(1).agecat ib(1).sex bmindx53

svy, subpop (if adultdepressionmono==1 & lowdosage!=.): logistic lowdosage chronicdz ib(1).agecat ib(1).sex bmindx53

svy, subpop (if adultdepressionmono==1 & lowdosage!=.): logistic lowdosage cancer ib(1).agecat ib(1).sex bmindx53

svy, subpop (if adultdepressionmono==1 & lowdosage!=.): logistic lowdosage pain age15x ib(1).sex bmindx53

svy, subpop (if adultdepressionmono==1 & lowdosage!=.): logistic lowdosage duration age15x ib(1).sex bmindx53

svy, subpop (if adultdepressionmono==1 & lowdosage!=.): logistic lowdosage ib(1).durationcat ib(1).agecat ib(1).sex bmindx53

svy, subpop (if adultdepressionmono==1 & lowdosage!=.): logistic lowdosage ib(1).antidepressanttype ib(1).agecat ib(1).sex bmindx53

//multivariable regression model

gen int_perwt15f=int(perwt15f)

//comprehensive + age as categ, income categ

svy, subpop (if adultdepressionmono==1 & lowdosage!=.): logistic lowdosage ib(1).agecat ib(1).sex ib(1).race ib(1).education ib(1).marital ib(1).famincomecat ib(1).inscov15 phq242 k6sum42 chronicdz cancer bmindx53 pain duration ib(1).antidepressanttype

logistic lowdosage ib(1).agecat ib(1).sex ib(1).race ib(1).education ib(1).marital ib(1).famincomecat ib(1).inscov15 phq242 k6sum42 chronicdz cancer bmindx53 pain duration ib(1).antidepressanttype [fw=int_perwt15f] if adultdepressionmono==1 & lowdosage!=.

estat gof

lroc

regress lowdosage ib(1).agecat ib(1).sex ib(1).race ib(1).education ib(1).marital ib(1).famincomecat ib(1).inscov15 phq242 k6sum42 chronicdz cancer bmindx53 pain duration ib(1).antidepressanttype [pw=perwt15f] if adultdepressionmono==1 & lowdosage!=.

estat vif
