# Supplementary figures and images for: National Prescription Patterns of Antidepressants in the Treatment of Adults With Major Depression in the US Between 1996 and 2015: A Population Representative Survey Based Analysis
Source: Front Psychiatry. 2020 Feb 14;11:35. doi: 10.3389/fpsyt.2020.00035 (PMC7033625; doi:10.3389/fpsyt.2020.00035)

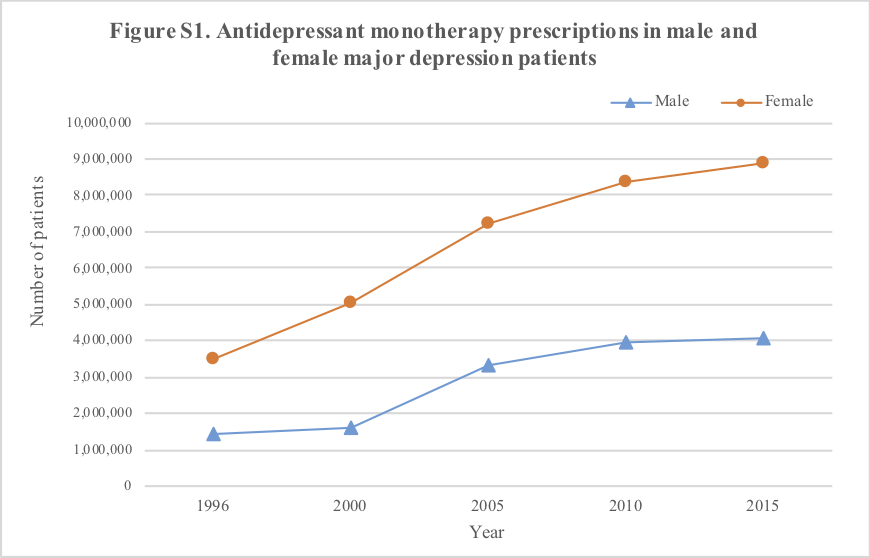

Supplement: Supplementary file 2 [file Image_1.tiff]

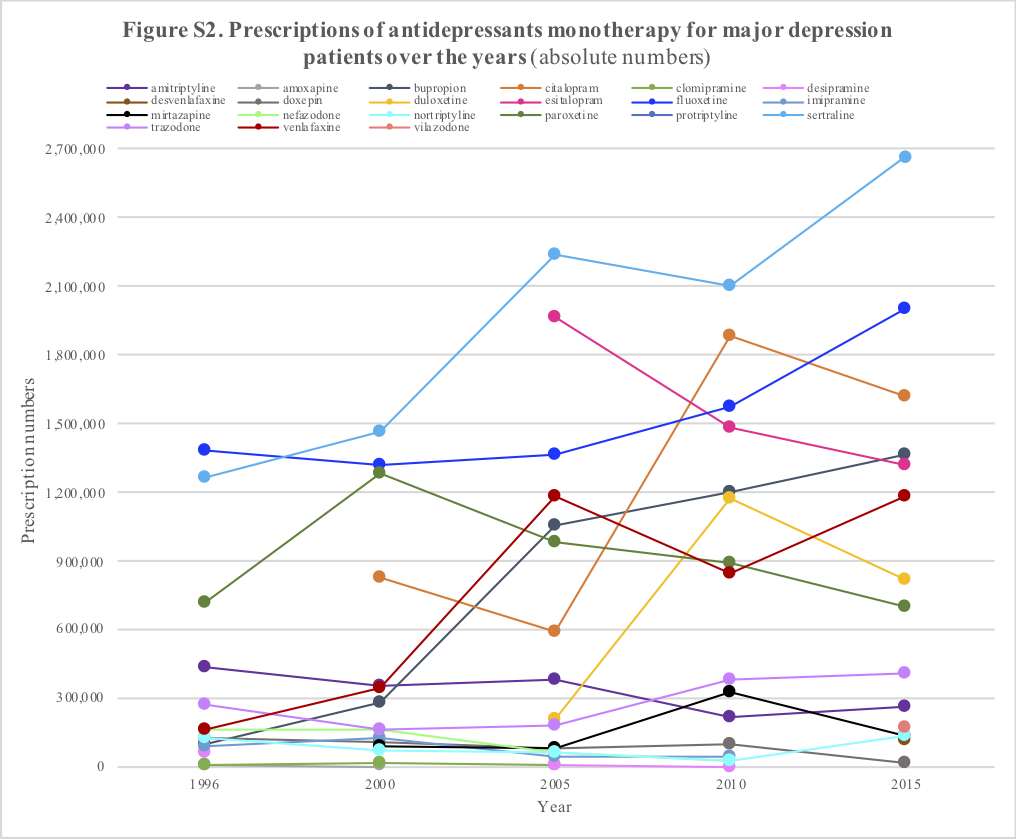

Supplement: Supplementary file 3 [file Image_2.tiff]

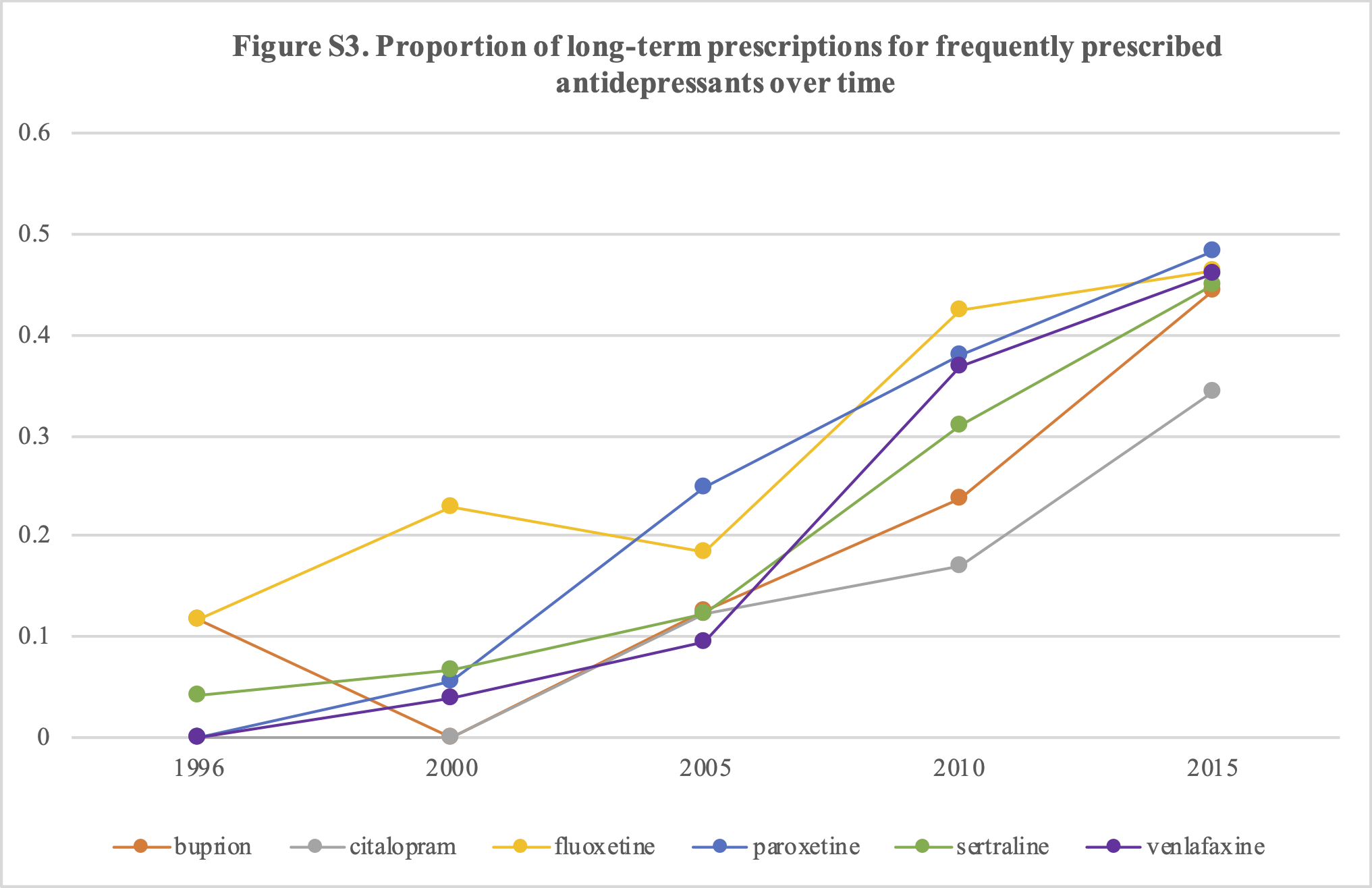

Supplement: Supplementary file 4 [file Image_3.tiff]
